# Supplementary material for: Whole-Genome Sequencing of Six Neglected Arboviruses Circulating in Africa Using Sequence-Independent Single Primer Amplification (SISPA) and MinION Nanopore Technologies
Source: Pathogens. 2022 Dec 8;11(12):1502. doi: 10.3390/pathogens11121502 (PMC9781818; doi:10.3390/pathogens11121502)
Supplement: Supplementary file 1 [file pathogens-11-01502-s001.zip › pathogens-2024600-supplementary.pdf]

Suppl. Table S1 RT-qPCR Protocol for MIDV and WSLV

S1.1 Primer sequences

	MIDV_PA_f	ACCATGCTAACGCGAGGGCGTTTTCGCA	
**MIDV**	MIDV_PA_r	CGGCGCGCTGCCTATRTCCAGGAT	
	MIDV_PA_p	TAATWGAAGGAGAGGTGGAAGTGGGC	FAM
	WESSV_f	GAAAGGAGTAGAAGAAAGGAGATTC	
**WSLV**	WESSV_r	TAGGTTCTTCACTCTAGCCGCTA	
	WESSV_p	CAACAAAGGGGATGAATAAGTCTCG	HEX

S1.2 Primer/Probe mixes

MIDV

16 µl	MIDV_PA_f (100 µM)
16 µl	MIDV_PA_r (100 µM)
2 µl	MIDV_p_Probe FAM (100 µM)
166 µl	0.1 x TE Buffer
200 µl	total

WSLV

16 µl	WESSV_f (100 µM)
16 µl	WESSV_r (100 µM)
2 µl	WESSV_p_Probe HEX (100 µM)
166 µl	0.1 x TE Buffer
200 µl	total

S1.3 Master-Mix

Processing steps	Master Mix	Volume
1.	**RNase free water**	3.5 µl
2.	**2× RT-PCR Buffer**	12.5 µl
3.	Primer-Probe-Mix 1: **MIDV-Mix FAM**	0.5 µl
4.	Primer-Probe-Mix 2: **WSLV-Mix HEX**	0.5 µl
5.	Primer-Probe-Mix 4: **b-Actin Mix Cy5**	2.0 µl
6.	25x RT-PCR Enzyme Mix	1.0 µl
	**Total Volume Master Mix**	**20 µl**
	**Total Volume**	**25 µl**

AgPath-ID™ One-Step RT-PCR Kit (Ambion)

S1.3 Cycler program

Cycler program			
**Reverse transcription**	50°C	20 min	
**Activation Taq**	95°C	15 min	
**Denaturation**	95°C	30 sec	45 cycles
**Annealing**	55°C	45 sec	45 cycles
**Elongation**	68°C	45 sec	

Suppl. Table S2 Generated genome sequences of all six viruses

>Crimean-Congo hemorrhagic fever virus segment L (animal origin)

TCTGCAGATACTCCCCGTTACCCACGCCAACACAGAGAGCTCCAGTAGTGGTCTTTCCCTTTG
CGAAACCATGGACTTCTTTAAGAAACCTTGATTGACTCGCGTGATTGCTGTCAATATGTGTCCA
ACCCTAGGGGTTCAACATTTGATTATTGAGATCGTGCGGCAGCCTGGTGATGGGAATCAAGCT
CGATCACAGCATAGCTGAGTTACCATGCCTAACAAAACAGATCACTCTCTATATCATATCGGT
CATCAAACGCGCCCAACCGAGTCGGCAGCACGGAAGTATTACCAGGAGGAGCCTGAAGCCA
GACTTGTTGGCCTGAACCTGGAAGATTATCTCAAGAGGATGCTGTCTGACAACGAGTGGGGAT
CAACTCCCTAGAAGCATCTATGTTGCCAAAGAAATGGGCATTACCATCATTATTTGACTGTTG
CCGCCAGTGATGAAGTGGAAGCAGGTTTAAAGTTCGAGACGATGCTTTACAGCTGTGAACCTT
TTGTACTCTGACAACAAACACACTTTGATGCGCTCAGAATACTGCCACAGTTTGAAAAACAGA
TACAAGAGAGGCCTTGAGCTTGATGGACAGGGTTATAGCTGTGGATCGCTAACATCATCTTCT
AGTGATGAACTGCAAGACTATGAAGACTTGCCTTGGCACTTACAAGCGCAGTAGCTGGGAAT
CAAATAGATTTCCGGTCAAGCTTCTTGGATGAGTCACATTGTCCAAGAAGCAAGCAGAGTTAC
TAAGGCAAAAAGCATCTCAGTTGTCTAAATTGGTCAATAAACAGAACATACCGACCACAGAG
TCGGAAAGAGTCTTGGATTGTATGTTCAACTGCAAATTATGTGTTGAGATATCAGCTGACACTT
TAATTTTGAACCAGACATGCCAAAAACAAATCAGAAATGACAGGTGAAAGCATGTCATCACG
ACAGTTGGGGCAACTTTAAACCGCTGACACGTAGACAAACAAAAGGTTAAGCAAGACTTCGT
CCAGAATGAAACTCTACGTCACTAAAGACTACTTTGCCTTGACCATCTAGCGACGTCCTGGTG
GGCTTTTGAGGGCTGCTTTCCCTGGAACAGGAATAGAGAGGCATATGCAGCTGCTACACTCTG
AGATGATACTGGACATCTGCACTGTATCACTTGGTGTCATGCTGTCTACATTCTTATATGGTTCT
AATAATAAAAACAAGAAGAAATTTATTACCAACTGTCTGCTCAGCACAGCCCTGTCCGGAAA
GAAGGTGTATAAAGTTCTCGGCAATCTAGGAAATGAACTGTTGTACAAGGCACCTAGAAAGG
CCTTAGCAACTGTCTGCAGTGCCTTGTTTGGTAAGCAGATAAACAAACTTCAGAATTGCTTCA
GGACCATAAGCCCTGTCAGCTTACTTGCATTGANAAATCTAGACTTTGATTGTCTCAGTGTAC
AAGACTATAACGGTATGATAGAAAACATGTCTAAATTAGACAACACTGATGTTGAATTCAAC
CACAGGGAGATAGCTGATCTCAATCAACTAACTTCTCGGCTTATCACATTAAGAAAGGAGAA
AGACACTGACCTCCTCAAACAATGGTTTCCTGAAAGTGACCTCACCCGCAGAAGCACCAGGA
ATGCTGCAAACGCGGAGGAATTTGTTATATCTGAGTTCTTTAAGAAGAAGGACATTATGAAAT
TCATCAGCACTTCAGGCAGAGCAATGAGTGCAGGCAAGATTGGTAATGTCCTATCCTACGCA
CATAATCTTTACTTGAGTAAGTCAAGCCTAAATATGACCTCTGAAGACATCTCACAGCTTTTG
ATCGAGATTAAGCGACTGTATGCTTTACAAGAAGATTCTGAAGTGGAGCCGATAGCCATAATT
TGTGATGGCATAGAAAGCAACATGAAACAGTTGTTTGCTATATTGCCTCCTGACTGTGCAAGA
GAGTGTGAAGTCCTCTTCGATGACATAAGAAATTCTCCAACACACAGCACAGCCTGGAAGCA
TACACTCCGATTAAAAGGGACTGCATACGAAGGTCTGTTTGCAAAGTGTTACGGCTGGCAATA
CATTCCAGAAGACATTAAACCAAGCCTGACCATGTTGATACAGACTTTGTTTCCTGACAAGTT
TGAAGATTTCCTGGATCGAACCCAGTTGCATCCGGAGTTCAGAGACCTGACTCCCGACTTTTC
GCTCACACAAAGGGTTCACTTTAAAAGAAATCGAATACCCAGTGTCGAAAATGTGCAAATCT
CCATTGATGCGACGTTGCCTGAATCTGTGGAAGCAGTGCCGGTGACAAGAAAGAAAGATGTT
CCACCTCTTCCTGAAACTCCGCTAAGTGNGGTGCATTCAATAGAGCGTATAATGGAAAACTTT
ACCCGCCTCATGCATGGAGGAGGGACTTTTGGCCAAGAAAAGAGAGTGGAGATCCGGCGAG
AACAGGGCAGCCAACAGAGTATCCACTGAACACGAGAGTTCTAGCATCTCTGCCTTTAAAGA
CTACGGAGAGAGAGGGGATAGTCGAGGAGAAATTCACAGTGCCAGGTTTAGTGAAGAAGAT
CAGCTAGAAACAAGNCAGCTGTTGTTGGTGGAAGCCTGGTTTCCAAACNGACATCGATGGGA

AAATAAGGACAGACCACAAGAAGTGGAAAGACATATTAAAGCTATGACAGCTACTAGGAAT
CAAGTGCTCATTTAGTTGCCTGTGCAGATTGCTCATCTACACCACCAGATAAATGGTGGATTTC
GGAGGACAGAGTGCGAGTCCTAAAAAATTCAGTCAGCTTCTTGTTCAATAAACTCTCTAGAAA
CTCACCTACAGAAGTAGCTGACATAGTTGTTGGAGCTATAAGTACTCAAAAGGTTANGAGTT
ACCTAAAGGCAGGAACTGCAACAAAAACCCCTGTGTCTACTAAAGACGTTCTGGAGACTTGG
GAAAAGATGAAGGAGCACATACTCAACAGGCCAACAGGACTGACACTGCCTACCAGTTTGG
AACAGGCAATGCGCAAAGGACTGGTCGAAGGTGTGGTCATCTCTAAGGAAGGTTCCGAGTCA
TGTATCAATATGTTGAAGGAAAATTTGGACCGAATAACTGACGAATTCGAGCGAACAAAATT
TAAACATGAACTTACTCAGAATATTACCACAAGTGAGAAGATGCTTTTGAGTTGGTTGAGTGA
AGACATCAAATCATCGAGATGTGGTGAGTGCCTCTTAAGTATGAAGAAAACTGTTGATGAAA
CTGCCAATCTATCAGAAAAGATTGAGCTACTCGCTTATAATCTGCAACTCACTAATCACTGCA
GCAACTGTCACCCCAATGGTGTAAGCATTAGTAACACTTCTAATGTGTGCAAGAGATGCCCCA
AGATTGAAGTGGTTAGCCATTGTGAAAATAAAGGCTTTGAGGACAGCAATGAATGTTTAACA
GACCTGGATAGGCTTGTTAGGCTCACATTACCAGGGAAAACTGAGAAGGAGAGAAGAGTCA
AACGTAACGTGGAATATCTTATAAAACTGATGATGAGCATGTCAGGCATTGATTGTATAAAAT
ATCCCACAGGGCAGCTTATCACCCATGGAAGAGTGAGTGCAAAAAATAACGATGGAAATTTG
AAAGATAGAAGCGATGACGACCAAAGACTAGCTGAGAAGATAGACACTGTTAGGAAAGAGC
TTTCAGAATCTAAACTGAAAGATTATTCAACTTATGCAAGGGGAGTGATATCAAATTCACTAA
AAAACCTCTCAAGGCAAGGTAAATCAAAGTGTTCTGTGCCAAGATCTTGGCTCGAAAAGGTA
CTGTTTGACCTGAAGGTACCTACTAAAGACGAAGAAGTACTTATAAATATCAGAAACTCATTG
AAAGCTAGATCCGAGTTTGTTAGAAATAACGATAAACTACTCATAAGGTCAAAAGAAGAACT
AAAAAAATGTTTCGACATGCAGTCTTTTAAATTGAAAAAAAACAAGCAACCTGTACCCTTTCA
GGTTGACTGTATACTGTTCAAAGAAGTGGCAGCTGAATGCATGAAGAGGTACATTGGCACAC
CTTATGAGGGAATTGTAGACACCTTAGTTTCTCTGATTAATGTGTTAACAAGGTTTACTTGGTT
CCAGGAAGTGGTCGCTAGATGGTCAAATATGTGAGGCCTTCCTAAGATGCTGCACAGAATTTA
ATAGGTCAGGGGTCAAGCTGGTTAAGGTAAGGCACTGTAGAGCATTAACCCTATCAGTTAAA
TTCTTGCCATCAAATAAAAAGGAGAATATGTTATTATTTGTATTGCCCACAAGGTTAATAGAA
ACACGACAGAATCGAACAGCACCTCCCAGACTCTGCAGATATCGGACCTTTTTCTGTATTTGA
ACAGGGAGACAAAGCTGTCAGCAAGTTCTTCAGTTACACCCTTTACATTGCTAAGGTTCATAG
GGAAACACTTTATATACAAGTGCTGCAGCACCTGCCGGGCTCTGCGGAAGATCACGCAGCTT
GGTACCAGGTGTCACAGAAGTTATAAACAGTGTGAGTGAAAAAACATTTGCAAGACATTGAA
AATCATTCCCATGACTCTACTAGAAGAAGATTGGAGCAAATTCAGGNAATACTCAGCTTTTGC
TCTTGAAGGTGGGATTTGAAGAATCATATAAAATACGAACCTCAAGGTGCAGAGCCGAGTGG
GAATTTTCTGAACAGGAGCAGTCAGAGACCACTTTATAAGTGTTGTTTCGCCCCCGATATCTG
GGCTCCTGAAGGTCCTAGTTCGGATTCTATCGTGCTTCCTATTGGCATAAAAGAACAACTTACT
AGACAACTCTCAGCAACAGAACAAACAGCTACAGATGCTTCGTTTGTAACATGCTGGCAGAG
CTAAATAGACTTATTTGGCCTAATGAGCTGCCAGGAAAGAAATTTTCGAATTGCGAAGCTGTA
GGAAAGAATTGAAGACAACATTGCAAGGCTTTACCTGCAGAACATCCATATACTGTTCTGTTA
GGGATGTGGAGGACAATGTTAAGCACTGGAAACAAAGAGATCTATGTCCTGAGGTAACCATT
CCATGCTTTACAGTCTATGGAACCTTTGTCAACAGCGATAGACAACTGATCTTTGACATTTACA
ATNTGCATATATATAATAAAGAAATGGACAACTTTGATGAAGGATGTATCAGCGTCTTGGAA
GAAACAGCAGAAAGACATATGCTTTGGGAACTCGATCTGTTGAACTCACTTTGTTCTGACGAA
AAGAAAGATACCAGAACCGCAAGACTATTACTAGGCTGCCCAAATGTGAGAAAAGCAGCAA
ACAAAGAAGGGAAGAAGCTGTTGAAGTTAAACAGCGACACATCCACAGACACACAGAGCAT
TGCTTCTGAAGTGTCGGACAGAAGTCTTATAGTTCAAGTAAGAGTAGAATCCGTAGTATATTT
GGTAGATACAACTCTCAGAAGAAACCATTTGAATTAAGGTCAGGTCTTGAGGTTTTCAATGAT

CCTTTCAATGATTACCAGCAAGCAATAACGGACATTTGCCAATTTTCTGAGTACACACCAAAC
AAAGAAAGCATTTTGAAAGACTGTCTTCAAATCATACGAAAAAACCCTAGCCACACAATGGG
TTCTTTTGAGCTGATCCAGGCAATCTCAGAGTTCGGCATGAGCAAGTTTCCTCCCGGAAAATA
TAGACAAAGCAAGAAGGGATCCGAAGAACTGGGTTAGCATCTCTGAAGTAACCGAAACAAC
AAGCATAGTTGCATCACCTAGAACTCATATGATGCTCAAGGATTGTTTTAAAATTATACTAGG
TATTGAGAATAAGAAGATAGTCAAAATGCTTCGAGGGAAGTTAAAGAAACTCGGTGCTATCT
CAACAAACATAGAGATTGGGAAAAGGGATTGCCTAGATCTACTTAGCACAGTAGATGGGCTA
ACAGACCAGCAAAAAGAAAATATTGTAAATGGAATATTTGAACCCTCAAAGTTATCCTTCTA
CCATTGGAAGGAATTAGTCNNAAAAAACATTGATGAAGTTTTACTTACTGAAGATGGAAATC
TGATCTTCTGCTGGTTGAAAACAATCTCCTCTTCAGTCAAAGGGAGCCTAAAGAAGAGACTCA
AATTCATGAATGTACATTCTCCAGAATTGATGCCGGAAAACTGTCTCTTTTCTAGTGAGGAATT
CAATGAGTTAATTAAGTTAAAGAAACTCCTCCTCAATGAACAACAAGATGAACAGGAGCTGA
AACAAGATCTTTTGATATCTTCTTGGATCAAGTGCATAACAGCTTGTAAAGATTTTGCAAGCAT
CAATGACAAGATTCAGAAGTTCATTTACCACCTGTCTGAAGAGCTATATGACATAAGGCTGCA
GCACCTGGAACTGTCAAAGCTTAAGCAAGAGCATCCTAGTGTCAGCTTCACAAAAGAAGAGG
TCTTAATAAAGCGGCTGGAGAAAAATTTCCTTAAGCAGCATAATCTAGAGATTATGGAAACT
GTGAATCTTATATTCTTTGCAGCCCTCTCAGCTCCTTGGTGCTTACACTATAAAGCACTAGAGT
CTTATTTGGTAAGACATCCAGAAATACTTGACTGTGGAACTAAAGAGGACTGCAAACTCACTT
TGCTTGATCCTGTCAGTTTCTAAGCTCTTGGTTTGTTTGTATCAAAAAGATGATGAGGAAGTGA
CGAATAGCTCAAGTTCCCTGAAACTTGGGTTTCTTAGTGAAATATGCTGTCACCTTGTTCACAC
ATCTATATGGCAAACCTTTTCAATCATATCTGCAATGAGCTCCGGCAGGTGCTGCTCGGATTCT
ATCGTATTTATGGCACAAGACTACTGACGAAAAGCCACTACATCAAACAAAGATAGTTTTTA
AAGCTAAAATTGGTTTATCTGGGAACAAAGTTATGACTTTATTCGGATCTTCATCTAAAAATG
ATAGCAACAGCGGTACCTTTTAACGTCTGCAAGAGATTAACGAAGAGTGACTGGGGAAAGGT
CTTGTAGCGCGTTAGCTTGTAGGTCATATGAGATGGTAGTGCTAGTGGGTGCTCCGGTGCATG
GCAATACTACGAACAAATTACCTATTGCATGGAAAGTACCAAATTAGGGAAGCACCGATACA
TATCCAAACTTAGCACCAAAAGCTCTGCAAATAACAGGAGCAAGAGATTTGTTAGTGCAGGG
AGACTGGGACTACTAAAGTTATGCATGCAAACCACTGAAATGTTTAGCAGAAATCTTTTTGAA
AACAACATCGGATGATGGCCTCACAAACCCACATCCCCGTGTGCAAAGAAACAATCCTCAAT
GTGGGATTAGACTGTCTTGCTAACATGCGAAATCTTGACGGTAAGCCCATAAGTGAAGGCAGT
AATTTGGTCAATTTTTACAAAGTCATATGCATCTCGGGTGATAATACCAAGTGGGGCCTGATA
CACTGCTGTTCTTTCTTTTCTGGCATGATGCAACAGGTTCTNAAAAATGTACCAGATTGGTGTT
CATTTTATAAATTAACATTCATAAAAAACTTGTGTAGACAGGTAGAAATACCTGCTGGCAGCA
TCAAAAAGATCTTAAATGTTCTTGAGATACAGATTGTGCAGCAAGGGAGGTGTAGAACAACA
CAGTGAAGAGGATCTGAGAAGACTGTTGACAGATAATTTAGACAGTTGGGACGGAAACGAC
ACAGTTAAGTTCTTAGTTACAACTTATATAAGCAAAGGTCTCATGGCGTTAAACAGTTACAAT
CATATGGGTCAGGGTATTCACCATGCACACATCTTCGGTGCTAACTTCCCTAGCTGCTGTGCTC
TTTGAGGAGCTGGCAANTTTTTATCTTAAGAGAAGCTTACCCCAGACAACAGTACATGTTGAA
CATGCCGGTAGTTCAGATGATTATGCAAAGTGTATAGTGGTGACTGGTACACTATCCAAAGAG
CTCTATTCCCAGTATGATGAAACATTTTGGAAACACTCTTGCAGACTCAAAAACTTCACGGCT
GCAGTACAAAGATGCTGTCAAATGAAAGATAGTGCCAAAACCTTGGTGAGCGACTGCTTTCT
CGAGTTTTACAGTGAGTTTATGATGGGTTACAGGGTAACCCCTGCTGTAATAAAGTTCATGTTT
ACTGGACTGATAAACAGCTCTGTGACCTCTCCTCAGAGTTTGATGCAAGCATGCCAAGTTTCA
TCCCAACAAGCAATGTATAATAGTGTTCCTCTTGTCACCAACACTGCCTTCACCCTATTAAGGC
AGCAAATCTTCTTTAACCATGTTGAAGACTTTATCAGAAGGTATGGTATACTGACTCTTGGGA
CTTTGTCACCCTTTGGTAGGTTGTTCGTACCAACCTACTCTGGATTAGTCAGCTCAGCAGTTGC

TTTAGAAGATGCTGAAGTCATTGCTAGGGCAGCCCAAACACTTCAGATGAACAGTGTGTCAAT
ACAGTCAAGTAGCTTGACCACATTAGATAGCCTAGGTCGTAGTCGGACAAGTTCCACAGCTG
AGGATAGCAGCAGTGTGAGTGATACTACTGCTGCTTCCCATGACTCAGGATCGTCATCTTCAA
GCTTCTCTTTTGAGCTCAATAGACCCCTGTCTGAAACTGAACTACAGTTTATTAAAGCACTAAG
TAGTCTCAAGTCAACACAAGCCTGTGAAGTGATTCAAAATAGAATTACAGGTCTTTATTGCAA
TAGCAACGAAGGACCTCTTGATAGGCATAATGTTATTTACAGCAGCAGAATGGCAGACTCTTG
CGATTGGCTAAAGGATGGCAAAAGAAGAGGAAATCTAGAACTAGCGAATAGGATTCAATCT
GTACTGTGTATTCTGATAGCAGGATATTACAGGTCATTTGGAGGGGAAGGAACCGAGAAACA
GGTAAAGGCATCATTGAATAGAGACGACAATAAAATCATAGAGGATCCTATGATACAACTAA
TTCCAGAAAAGCTGAGGAGAGAGTTAGAAAGGTTAGGTGTTTCTAGAATGGAGGTCGATGAG
CTAATGCCAAGCATTAGTCCTGATGACACTTTAGCCCAGCTTGTAGCAAAAAAACTCATCAGT
CTCAATGTTTCGACAGAAGAATACTCAGCTGAAGTGTCTAGACTCAAACAGACACTGACAGC
CCGAAATGTTTTGCACGGGTTAGCTGGAGGAATTAAGGAGCTTTCGCTTCCAATATATACAAT
ATTCATGAAATCTTACTTCTTTAAAGACAATGTCTTCCTGTCACTAACAGATAGATGGTCTACC
AAGCACAGTACAAACTATCGTGATAGTTGTGGCAAACAATTAACAGGTAGAATAATTACCAA
GTATACTCACTGGTTGGACACTTTTCTGGGCTGCTCTGTCTCCATCAACAGGCATACCACCGTT
AAAGAGCCCTCCTTATTCAATCCGAATATCAGATGTGTGAATCTGATCACATTTGAGGACGGC
CTGAGAGAACTGTCAGTGATACAGAGTCACCTTAAAGTCTTTGAAAATGAGTTCACCAACTTA
AATCTTCAATTCTCTGATCCGAACAGACAGAAACTTAGAATAGTTGAGTCCAGACCTGCAGA
ATCCGAGCTAGAGGCAAACCGTGCAGTGATTGTCAAGACCAAATTGTTTCAGCAACTGAAC
AAGTTCGGCTATCCAACAACCCTGCAGTTGTCATGGGCTACCTATTGGATGAATCTGCAATTT
CTGAAGTCAAGCCTACCAAGGTTGACTTCTCAAATTTGCNNAAAGACCGCTTCAAAATAATG
CAATTTTTCCCTTCAGTGTTCACTTTAATTAAGATGCTGACAGATGAATCGTCAGATTCAGAAA
AGAGTGGCCTTAGTTCAGATTTGCAACAAGTTGCAAGATACTCAAACCACTTGACCTTGCTCA
GCAGAATGATTCAACAAGCAAAGCCAACCGTGACTGTTTTCTACATGCTAAAAGGTAACTTG
ATGAACACAGAGCCAACAGTTGCTGAGCTTGTCAGCTATGGTATAAAGGAAGGCAGATTTTTT
AGGCTTTCCGACACCGGAATCGATGCAAGCACATACTCTGTAAAATATTGGAAAATTCTTCAC
TGCATCTCTGCCATTGGATGTCTACCTTTGAGCCAAGCAGACAAGTCTTCACTACTTATGAGCT
TCTTAAACTGGAGGGTCAACATGGACATTAGAACATCTGACTGTCCACTGTCTAGTCATGAAG
CAAGTATATTGAGTGAATTTGATGGACAAGTCATCGCTAACATACTTGCCAGTGAATTAAGTT
CTGTGAAACGAGATTCTGAACGCGAGGGTCTGACTGATCTCCTTGATTATCTAAACTCACCAA
CTGAATTGTTGAAGAAAAAGCCTTACTTAGGGACAACGTGCAAGTTCAACACCTGGGGAGAC
TCGAATAGATCTGGAAAGTTCACATACAGCAGCAGATCTGGAGAATCCATTGGAATCTTCATT
GCAGGGAAACTGCACATCCATCTCTCATCTGAGTCCATTGCCTTGTTGTGTGAAACTGAAAGA
CAAGTGCTTTCTTGGATGAGCAAGAGGAGGACTGAGGTAATAACTAAAGAACAGCACCAACT
GTTTTTAAGTCTTCTCCCACAGTCTCATGAGTGTTTACAAAAGCACAAAGACGGAAGTGCACT
ATCAGTCATACCTGATGGCAGCAATCCTCGATTACTTAAGTTTGTGCCCCTCAAAAAAGGTCT
AGCAGTGGTGAAAATCAAAAAACAAATTTTAACAGTGAAGAAGCAGGTTGTGTTTGATGCAG
AGAGCGAGCCTAGACTGCAGTGGGGGCATGGCTGCTTGTCCATTGTTTATGACAGAAACTGAT
ACTCAGACCACATACCATGAAAATCTCTTGAAGGTGAAGCATCTTGTTGACTGCTCTACAGAT
AGGAAGAAGCTTTTGCCCCAGTCAGTGTTTTCTGACTCCAAAGTTGTCCTTTCAAGGATCAAGT
TCAAGACGGAGCTTCTCCTCAACTCATTGACGCTGCTCCACTGTTTCCTAAAACATGCTCCTAG
TGATGCCATAATGGAGGTAGAGAGCAAAAGTAGCTTACTACACAAGTACCTCAAATCGGGAG
GTGTCAGGCAACGGAACACTGAAGTGCTCTTCAGAGAGAAGTTAAACAAGGTTGTTATAAAA
GACAATCTTGAGCAAGGTGTGGAAGAAGAGATTGAGTTTTGCAACAACTTGACTAAGACTGT
TTCAGAGAACCCATTACCACTTAGCTGTTGGTCTGAAGTTCAAAATTACATTGAAGACATAGG

CTTCAACAATGTTCTTGTTAACATTGACAGAAACACGGTGAAAAGTGAACTTTTATGGAAATT
TACGTTAGACACCAATGTAAGCACCACAAGTACTATAAAAGACGTGAGGACATTGGTGTCCT
ACGTTAGCACTGAAACCATCCCTAAGTTCTTGCTTGCATTCTTACTTTATGAGGAGGTGTTAAT
GAACTTGATCAACCAGTGTAAGGCAGTAAAAGAACTCATCAACAGCACAGGACTCTCAGACT
CGGAACTGGAAAGCTTACTCACTTTATGTGCTTTCTATTTCCAAAATGAGTGCAGTAAGAGAG
ATGGCCCTAGATGCTCCTTTGCAGCACTATTAAGTCTAATCCATGAGGAATGGCAGAGGATAG
GTAAAAACATTCTTGTTCGTGCAAACAATGAACTAGGTGATGTGTCACTTAAGGTTAACATTG
TCTTGGTGCCTCTCAAGGACATGTCTAAGCCAAAGTCTGAGAGAGTGGTCATGGCCAGAAGG
TCACTAAATCATGCTCTATCCTTGATGTTTTTAGACGAGATGTCATTACCTGAACTAAAATCCC
TATCCGTGAACTGCAAAATGGGGAACTTTGAAGGGCAGGAGTGCTTTGAGTTCACTATTCTGA
AGGACAATAGCGCAAGGCTAGATTACAACAAGTTGATTGACCACTGTGTGGACATGGAAAAA
AAGAGGGAAGCGGTTAGAGCAGTAGAGGATTTAATTTTAATGTTGACAGGCAAAGCAGTCAA
ACCCAGTGCTGTGACACAGTTTGTACACGAGGACGAGCAGTGTCAAGAGCAAATAAGCTTAG
ATGATCTGATGACAAACGACACGGTTACAGACTTTCCTGATAGGGAAGCAGAAGCCCTCAAA
ACAGGAAATCTTGGCTTTAACTGGGATTCAGATTGAATATACC>

Crimean-Congo hemorrhagic fever virus segment M (animal origin)

TCTGNAGCGACACTACATGCGGCACGTCAGTACGTAAGTGTTAAGACTACTTTGGGTGTGTGT
GAACAGCGATTTGTGAGGAACATGTTTCTCCACTTCAAATTTATGTTGTTGACTAACTTCTTCTT
TTGTCAACTCCTATGGGGTGGCGGCAGGGGTTGCCAGTGGAGGCAGGACCAATACTAGCTCA
ACACAAACTACCCTAACATCTTCAAACACTTCAAATAGTACAATGTCTGTGCTGGAGTCATCA
ATAGGAACTGCCACAGAAACAGTGCCGAGCACGACCACCAACACACCGGACACACAGGTCA
CTACAGACAGTGGTTCTGGTGAATCCAGCACAGACCCCCAGACCACCAGCANNGAAGTACCT
TCCACTTCTGACGCCACCATCAAAACACAAGAGTCAGAAACGCTCAGGGCTCAAAACCCAAG
TACTACGGCTGGCTCAGGTGCATCCACTCCAAGCAACACCACATCAAATAAACCAGATGTCA
ACTCACCCACATCACCAATCTTGGAGGCAAAGTCATCACCCAGCCCTAGTAAAACATCGACG
CCACAGACTGCACATCATCTCTCCAGAAGTCTTCTCTCAGCAGCAACTACAGAAACAGATCA
AACAGCATCAGTAATGACTACAACTAGAGAGACAACAACCATGAGCAGTCCAACAACAGTC
AAAAACAGTGTAACAGCCATACCAACCCAAGGCATCACCACCAGCAACACTTCGAAACAGA
TTAACACTACAACCCTAGGGACTCCCCTAAGATTCAAGCAACACAGAGTCAACAGACTGAAA
CACCAGCAATCGCACCTGCCACAACTGCTATTGCATTCATAAGCCCTACAAACAGATCTAAA
AGGGAATCCAAGGTGCAAATAATTCTGACTTTCTCTCAAGGCCTCAAGAAATATTATAGCAA
GATACTGAAACTCCTACACCTGACACAAGAAGAAGATTCTGAAGGTCTGCTTGAATGGTGCA
CGCGGGTACTTGAACAGGCATGTGATGACGACTACTTCAATGAAAGAATAGGAGAATTTTTC
ATAACCGGAAAAGGCTACTTCAATGATGTTTTGCAGTTTAGGCTGTATGACACACTTAGTACC
ACCGAATCAACCCAGAACACCTCACCCACAGCCAAGCCTTTCAGGTCATACTATGCCAAAGG
GTCTCTTACCTTTGACTCAGGCTATTTCTCTGCTAAATGTTATCCAAGGGCATCTAACTCAGGA
TTGCAGCTAATTAATGTCACACAACATTCAGTGAAAATAGCTAACACGCCAGGCCCCAAAAT
ATCTAATCCTAAGACCATCAACTGTATAAACTTGAAAGTGTCAACTGACAAGGATCATAGCA
AGCTTGAGATCAACGTACTTCTACCACAAGTTGCTGTTAATCTTTCAAACTGCCATGTTTTAAT
TGAATCACATGTCTGTGATTACTCTTTGGACACTGATGGGACAATAAAGCTCCCAAAAATTGC
ACATAATGGGGCTTTTATACCAGGTACTTACAAAATTGTGATAGATAAGAAAAACAAATTAA
ATGATAGGTGTGCACTATTCACCAGCTGCGTCATCAAAGGAAGAGAGGTCCGAAAAGGGCAG
TCAGCTCTGAGGCAGTACAGAACAGAGATAAGAATCGGGCAAACATTTACAGGCTCGAGGA
GATTGCTTGCAGAGGGGGGAAGCAACGATTGTGTTTCAAGAACCCAGCTAGTCAAAACAGAG

GTTGCAGAAATTCATGAAGACAGTTACGGTGGACCTGGCGAAAAAATCACAATTTGCAACGG
CTCCACAATAGTAGATCAGAGGCTTGGCAGCGAATTAGGTTGCTATACAATAAACAGAGTGA
GAACCTATAAGCTCTGTGAGAACAGTGCCACAGAAAAGAGCTGTGAAATAGACAGTGTTCCA
GTTAAATGCAAACAGGGGTTCTGCCTCAAAATAACTCAGGAAGGTAGGGGCCATGTGAAATT
GTCTAGAGGGACAGAAATCGTTTTGGATGTCTGTGATACTAGCTGTGAACTGATGATACCCAA
AGGCACTGGGGATATATTGGTGGACTGCTCAGGAGGGCAGCAACACTTTTTACAGGACAATC
TAATTGACTTGGGATGCCCAAATATTCCATTGCTGGGTCAAACAGCAATATATGTTTGTAGAA
TGTCAAACCATCCCAAGACAACCATGGCTTTTCTGTTTTGGTTCAGTTTTGGCTATGTCATCAC
ATGTGTAGTGTGCAAGGTGCTCTTCTATTTGCTAATTGTCATCGGGACACTAGGAAAAAAGCT
GAAACAGTACAGAGAACTAAAGCCTCAGACATGTATTGTCTGTGAATCTATCCCGGTCAATG
CAATAGATGCTGAAATGCACGATCTTAACTGCAACTACAACATATGCCCTTACTGTGCATCCA
GACTGACCTCAGATGGTCTCGTCAGGCACGTAACTCAATGTCCCAAACGGAAAGAGAAGGTC
GAAGAAACTGAACTATACTTAAACCTGGAGAGAATACCTTGGTTGGTGAGAAAACTCCTACA
AGTGTCGGAGTCAACAGGAGTGGCTCTGAAGAGAAGCTGCTGGATGATCACACTCCTTATCCT
GCTGACTGTTTCAATGTCACCAGTTCAGTCCGCACCAGTAGGCCACAAAAGGGCAGTTGAAG
TCTATCAAATGAGAGAAGGCTATACAGGTATTTGCCTCTTCGTGTTGGGAAGTGTTCTCTTCGC
AGTCTCTTGGCTGGTGAAGGCCCTGATTGATAGCATCGGCAATAGTTTCTTCCCTGGGCTTTCC
ATATGCAAGACGTGTTCCATTGGTAGTATAAATGGATTTGAAATAGAGTCACACAAATGCTAC
TGCAGTCTATTTTGCTGTCCTTACTGTAGGGCCTGCTCATCGGACAAAATCACTCACCAAATGC
ATTTGAATGTGTGCAAAAAGAGAAAGGTGGGTAGTAATGTTATGCTAGCCGTTTGCAAACGC
ATGTGTTTTAAAGCAACTATTGAAGCAAGCAACAGGGCCACTTTCATCAGAAACATCATAAA
CTCCACTTTTGTCATATGCATACTAATCCTGGTGATCTGTGTGGTTAGTACCTCTGCTGTCGACA
TGGAAAATTTACCGGCAGGCGTTTGGGAAAAAGAGGAAGACCTAACAAACTTCTGCCATCAG
GAGTGCCAAGTAACAGAAACTGAATGCCTTTGCCCATATGAAGCCTTAATGCTTAGAAAGCC
ACTTTTCTTAGATAGTATAGCCAAGGGAATGAAAAGCTTGTTGAACTCCACAAGTTTAGAAAC
GAGCTTGTCAATTGAAGCTCCGTGGGGGGCAATCAATGTTCAATCAACTTTCAGACCAACAGT
TTCAGCCGCCAATATAGCACTTAGCTGGAGTTCAGTTGAACACAAGGGCAACAAGATCTTAG
TTTCAGGTAGATCAGAGTCAATTATGAAATTAGAAGAAAGAACTGGAATCAGCTGGTGTCTA
GGTGTGGAAGATGCCTCTGAGTCAAAAACACTCACCGTCTCTGTTATGGATCTGTCTCAAATG
TATTCTCCTGTTTTCGAGTACCTGTCAGGTGACCGACAGGTGGAGGAGTGGCCAAAAGCAACC
TGCACAGGTGACTGTCCAGAAAGATGTGGCTGCACTTCATCAACCTGCCTGCATAAGGAGTG
GCCGCACTCGAGGAACTGGCGCTGTAACCCCACTTGGTGTTGGGGGGTGGGAACTGGCTGCA
CCTGCTGTGGGCTAGATGTAAAGGATCTCTTTACAGACTACATGTTGGTTAAATGGAAAGTGG
AATATATAAAAACAGAAGCCATAATATGTGTTGAGCTTACTAGTCAAGAGAGGCAATGTAGT
TTAATTGAAGCAGGCACAAGGTTTAACCTAGGCCCTGTGACAATAACTCTATCAGAACCAAG
GAACGTACAGCAGAGGCTTCCTCCCGAGATCATCACACTGCACCCTAAGATAGAAGAAGGGT
TTTTTGATTTGATGCATGTGCAGAAGGTAATGTCTGCAAACACGGTTTGTAAGCTGCAAAGCT
GTACTCATGGTGTGCCTGGAGACTTACAAGTCTACCATGTTGGCAACTTGTTAAAGGGGGACA
AAGTCAATGGGCACTCAATCCATAAGATAGAATCACATCTCAACACCTCATGGATGTCATGG
GATGGGTGTGATTTAGACTATTTTTGCAACATGGGGGACTGGCCCTCATGCACATACACAGGG
GTGACACAACACAACCATGCTGCATTCATCAACATGCTTAACATTGAGACTGATTACACAAA
AAACTTTCACTTTCATTCAAAAAGGGTCACAGCACACGGAGACACCCCTCAGTTGGACCTAA
AAGCGAGACCAACATATGGTGCAGGCGAAATCACTGTTCTAGTAGAAGTTGCAGATTTAGAA
CTGCACACGAAGAAAATTGAAGTGTCGGGTCTGAAATTCGCAAGTTTGTCATGCTCCGGATGT
TATGCTTGCAGCTCCGGCATCTCCTGCAAAGTTAGGATTCATGTAAATGAACCGGATGAATTC
ACAGTACATGTAAAAAGTAATGACCCAGATGTTGTAGCAGCAGGATCAAGTCTCATGGCTAG

AAAGATAGAGTCTGGGGCCGACAGCACGTTTAAGGCTTTTTCATCAATGCCAAAAAACTCTCT
ATGTTTTTACATAGTAGAAAGAGATTATTGTAGCAGCTGTACAGATGATGACACTCAGAAATG
TGTTGACATTAAACTTGAACATCCACAGAGCATACTCATTGAACACAAAGGAACGATTATTG
GCAAGCAAAATGACACCTGTCCAACTAAAACAAGCTGCTGGTTGGAATCTATTAAGAGTTTTT
TTNATGGGCTTAAAAACATGCTGAGTGGGATCTTCAGTAATGTTTTCTTAGGCATGCTCCTGTT
TCTTGCTCCTTTTGTATTGCTAATATTGTTTTTCTTCTTCGGGTGGAGACGTCGCTAGATGCCTTA
AGTGCTGTAGAAGGACCAGAAAGCTGCTAAAGTATAAGCACCTTAGGGATGCGAAGAAGAG
AGAGTGGTTACAAAAAAATCATTGAAAGGCTAAATGACCACCAAAAAAGGCAAAAGTCGAT
TGTTTGATGGAGAAAGGCTTGCAGACAGAAAAATTGCCGAACTCTTTTCAACTAAAACTCATA
GGTTGACGAATCAGACAAGCTGTTTTCTGCCCACCAATATTCATCTTTAATGTTGCTACATACA
TCTTCCCTAACCAAACATGCCCCAATTATCACATACCTGAACAGTGGAACTACCTTAGATGAT
AACACACACCTGCTTGGAACTCGCAACAGGAACGCAGAAAACGTTAGTGGATTAACGCTCTT
AATATCGTGTTTCCCTATTAACTTAGCAATACG

>Crimean-Congo hemorrhagic fever virus segment S (animal origin)

TGTTCACTTCGATTTTGTTCTCCATTGTTGCCTTGAGTGTTAGCAAAATGGAGAACAAAATCGA
AGTGAACAACAAAGATGAGCTTAACAAATGGTTTGGGAGTTCAAGAAAGGAAACGGGCTTGT
GGACACTTCACAAACTCCTATTCCTTCTGTGAGAACGTGCCAAACCTGACAGGTTTGTATTCC
AGATGGCTGGTGCAACCGATGATGCACAAAAAGACATTCCATCTATGCATCTGCCAAGGTTTT
ATGAGGGAAACACCATAGAACTGTGCTCCTGATAGGCATGCACCTCGGGCCTGCGTTAGCCC
CACAGATATANTNAAAAGAGGCCTTGAGTGGTTCGAAAAAAAACACAGGAACCATTAAAAG
TCTCTGGGATGAGAGCTACACTGAACTACTGGAAAGCTTGATGTGCTCCAAAATTTCGAACAG
CTTGCCAACTACCAGCAGGCTGCTCTCAAGTGGAGGAAGGACATAGGGCTTCCGCGTCAATG
CAAAACACAGCAGCCCTAAGCAATAACAAAGTCCTCTCTGAGTACAAGGTTCCTGGGTGAGA
TTGTAATGTCTGTCAAAGAGATGCTTTCAGACATGATCAGAAGGAGGAACCTCGATCTCTCAA
CAGAGGGGGTGATGAAAACCCAAGGGGCCCAGTAAGCAAGGAGCACATAGAATGGTGCAG
AGAGTTTGTCAAGGGCAAATACATAATGGCCTTCAATCCACCCTGGGGTGATGTCAACAAGTC
CGGCCGCTCAGGAATAGCACTAGTTGCTACAGGTCTTGCCAAACTTGCAGAAACAGAGGGAA
AGGGAGTTTTTGAGGAGGCCAAAAAGACGGTGGAGGCCCTCAAGGAGTACCTTGACAAACA
CAAAGATGAGGTAGACAAGGCCAGTGCTGATAGCATGGTAACAAACCTCCTCAAGCACATCA
CCAAGGCCCAGGAACTCTACAAGAACTCATCAGCACTGAGAGCACAGGGTGCACAAATTGA
CACCCCTTTCAGCTCCTTCTACTGGCTCTACAAAGCGGGCGTGACTCCAGAAACCTTCCCCAC
TGTCTCTCAATTCCTNTTTGAGCTGGGAAAGCAGCCAAGAGGCACTAAAAAANTGAAGAAGG
CACTTCTGAGCACCCCAATGAAGTGGGGAAAGAAGCTTTATGAGCTCTTTGCTGATGATTCTT
TCCAGCAAAACAGGATCTACATGCACCCTGCTGTATTGACAGCTGGCAGAATCAGTGAGATG
GGTGTCTGCTTTGGGACCATTCCTGTTGCCAATCCGGACGATGCAGCCCAGGGCTCGGGCCAT
ACCAAGTCAATTCTGAACCTCCGAACAAATAGTGAAACTAACAATCCCTGTGCCAAAACGAT
TGTCAAGCTTTTCGAGGTTCAGAAAACAGGATTCAACATACAGGACATGGACATTGTTGCCTC
TGAGCATCTGCTGCACCAGTCTCTTGTTGGCAAGCAGTCCCCGTTCCAAAATGCTTACAACGT
CAAAGGCAATGCCACCAGTGCCAACATCATCTAAATCTCCAAAATTTTTTTTTATTTGTTCCAG
TTTGTGCTTCTGCTTCTAACCACAACTAACAACCGCATTTGCTTTTTNNAGTTACCAAAACCTA
TTTTATCTTGCTTTATTTCATTTTACTTCATGCTATTATATTCTTCTTTTACATATTAAAGGGCTGT
GCGGCAACGATATCTTGCAGA

>Rift Valley fever virus segment L (animal origin)

ACACAANGGCGCCCAATCATGGATTCTATATTATCAAAACAGCTGGTTGACAAGACTGGTTTT
GTTAGAGTGCCAATCAAGCATTATGACTGTACAATGCTAACTCTGGCACTCCCAACATTTGAT
GTCTCCAAGATGGTAGATAGAATTACCATAGACTTCAATTTAGACGACATACAAGGAGCATC
TGAAATAGGCTCAACTTTGCTACCCTCTATGTCGATAGATGTGGAAGATATGGCCAATTTTGTT
CACGATTTCACCTTTGGCCACTTAGCTGACAAGACTGACAGACTCTTAATGCGTGAGTTTCCC
ATGATGAATGACGGGTTTGATCATCTGAGCCCTGACATGATTATCAAAACTACATCTGGCATG
TATAACATCGTTGAGTTCACCACCTTTAGGGGGGATGAAAGAGGTGCATTCCAGGCTGCCATG
ACTAAACTCGCTAAGTATGAGGTTCCTTGTGAGAACAGATCTCAGGGCAGGACTGTTGTTCTT
TATGTTGTTAGCGCCTACCGGCATGGTGTTTGGTCTAATTTGGAGCTAGAGGACTCTGAAGCA
GAGGAGATGGTATATAGGTACAGACTTGCCCTTAGTGTGATGGATGAGCTAAGGACCTTGTTC
CCAGAACTGTCATCCACAGATGAGGAACTAGGAAAGACTGAGAGAGAGTTGCTAGCCATGGT
CTCCTCCATCCAAATAAATTGGTCAGTCACAGAATCTGTGTTTCCTCCCTTTAGCAGAGAAATG
TTTGACAGGTTCAGATCTTCTCCTCCCGATTCAGAGTACATCACGAGGATAGTGAGCAGATGC
CTCATAAATTCTCAAGAGAAACTCATCAATAATTCCTTCTTTGCTGAAGGGAATGATAAAGTT
TTGAGATTTTCAAAAAACGCTGAGGAGTGTTCCTTGGCAATAGAGAGAGCTTTAAATCAGTAT
AGGGCAGAAGACAACCTTAGGGACCTAAATGACCACAAGTCTACTATTCAGCTGCCTCCCTG
GCTGTCCTATCACGATGCCGATGGCAAAGATCTGTGCCCTCTTCAGGGATTAGATGTGAGAGG
AGACCATCCCATGTGCAACCTGTGGAGAGAAGTGGTTACCTCTGCAAATCTAGAGGAGATTG
AGAGGATGCACGATGATGCAGCGGCAGAACTTGAGTTTGCCCTTTCAGGGGTGAAGGACAGG
CCAGATGAAAGAAACAGATACCATAGAGTCCATCTGAATATGGACTCAGATGATAGTGTCTA
CATAGCTGCTTTAGGGGTTAATGGAAAGAAGCATAAAGCAGACACATTAGTGCAACAAATGA
GAGACAGGAGCAAACAGCCCTTCTCTCCAGATCATGATGTGGATCACATATCTGAATTTCTCT
CTGCATGCTCTAGTGACTTGTGGGCAACAGATGAGGACCTATACAACCCTCTCTCTTGTGATA
AAGAGCTTAGATTGGCAGCTCAGAGAATTCATCAGCCATCCTTATCAGAAAGGGGCTTCAAT
GAGATTATAACAGAGCACTACAGATTTATGGGAAGTAGGATAGGATCATGGTGCCAAATGGT
CAGTTTAATAGGAGCTGAGCTATCAGCTTCTGTAAAGCAACATGTTAAGCCTAACTATTTTGT
GATTAAACGACTACTAGGTTCTGGGATTTTCTTGCTGATCAAGCCTACTTCCAGCAAAAGCCA
TATATTCGTGTCTTTTGCAATTAAGCGCTCTTGCTGGGCCTTTGATCTCTCCACTTCCAGGGTTT
TCAAACCCTACATAGATGCCGGGGATCTGTTAGTTACTGACTTTGTTTCTTACAAACTAAGTAA
GCTTACCAACCTCTGCAAGTGCGTTTCGTTAATGGAATCCTCCTTCTCATTTTGGGCAGAGGCA
TTTGGGATTCCAAGCTGGAACTTTGTTAGTGACTTGTTCAGGTCTTCAGACTCTGCAGCAATGG
ATGCCTCATACATGGGCAAACTCTCTTTATTAACCCTTTTGGAAGACAAAGCAACAACTGAAG
AGTTACAGACTATTGCAAGATATATAATCATGGAGGGCTTTGTCTCGCCCCCAGAAATCCCAA
AACCTCACAAGATGACCTCTAAGTTTCCCAAGGTTCTCAGGTCAGAGCTGCAGGTTTACTTAT
TAAACTGCTTATGCAGAACTATCCAGAGAATAGCAGGTGAGCCCTTTATTCTTAAGAAGAAG
GATGGGTCTATATCCTGGGGTGGCATGTTTAATCCTTTTTCAGGGCGTCCACTGCTTGATATGC
AACCACTCATCAGCTGTTGTTACAATGGTTACTTTAAAAACAAAGAAGAAGAGACTGAGCCT
TCCTCCCTTTCTGGGATGTATAAGAAAATTATAGAACTTGAGCACCTTAGACCACAGTCAGAT
GCCTTCTTGGGTTATAAAGATCCAGAACTACCTAGAATGCATGAGTTCAGTGTTTCCTACTTGA
AGGAGGCTTGCAATCATGCTAAGCTGGTCTTAAGGAGTCTCTATGGACAGAATTTCATGGAGC
AAATAGACAACCAAATTATTCGAGAGCTCAGTGGGTTGACTCTAGAAAGATTAGCCACACTT
AAGGCCACAAGCAACTTTAATGAGAATTGGTATGTCTATAAGGATGTGGCAGACAAGAACTA
CACAAGGGATAAATTATTAGTGAAGATGTCAAAATATGCTTCTGAGGGAAAGAGCCTAGCTA
TCCAGAAGTTTGAGGATTGCATGAGGCAGATAGAGTCACAAGGATGTATGCACATTTGTTTGT
TTAAGAAACAACAGCATGGAGGTCTGAGAGAGATCTATGTGATGGGTGCAGAGGAAAGAAT
TGTTCAATCGGTGGTGGAGACAATAGCCAGGTCTATAGGGAAGTTCTTTGCTTCTGATACCCTC

TGTAACCCCCCCAATAAGGTGAAAATTCCTGAGACACATGGCATTAGGGCTCGGAAGCAATG
TAAGGGGCCTGTGTGGACTTGTGCAACATCAGATGATGCAAGGAAGTGGAACCAAGGCCATT
TTGTTACAAAGTTTGCCCTCATGCTATGTGAGTTCACCTCTCCTAAGTGGTGGCCATTGATCAT
TAGGGGATGTTCAATGTTTACCAGGAAAAGGATGATGATGAATTTGAATTATCTTAAGATCCT
GGATGGTCATCGAGAGCTTGATATTAGAGATGACTTTGTGATGGATCTCTTCAAAGCTTATCAT
GGTGAGGCAGAAGTTCCATGGGCTTTTAAGGGTAAAACATATCTGGAAACCACGACAGGGAT
GATGCAGGGGATATTGCATTATACTTCCTCATTATTACACACCATTCATCAAGAATACATCCG
GTCCTTGTCCTTTAAAATATTCAACCTGAAGGTTGCTCCTGAGATGAGCAAAAGCCTGGTTTGT
GACATGATGCAAGGATCAGATGATAGTAGCATGCTAATCAGCTTCCCAGCTGATGACGAGAA
GGTTCTCACCAGATGCAAAGTGGCCGCAGCCATATGCTTCCGAATGAAGAAGGAGCTGGGAG
TGTACCTTGCCATCTACCCCTCAGAGAAGTCCACAGCAAACACAGATTTTGTGATGGAGTACA
ATTCTGAATTTTATTTCCACACCCAGCATGTTAGACCGACGATCAGGTGGATTGCAGCATGTTG
CAGCCTGCCAGAAGTGGAAACACTAGTAGCCCGCCAGGAAGAGGCCTCTAATCTAATGACTT
CAGTTACTGAGGGGGGTGGGTCATTCTCCTTAGCTGCAATGATTCAGCAAGCTCAGTGCACTC
TCCATTACATGCTAATGGGCATGGGAGTGTCTGAGCTATTCTTAGAGTATAAGAAGGCAGTGC
TGAAGTGGAATGACCCTGGTCTGGGTTTCTTCCTGCTTGACAATCCTTATGCGTGCGGGTTGGG
AGGTTTTAGATTTAATCTCTTCAAAGCCATCACCAGAACTGATTTGCAGAAGCTATATGCTTTC
TTCATGAAGAAGGTTAAGGGCTCAGCTGCTAGGGACTGGGCAGATGAGGATGTTACCATCCC
AGAAACGTGTAGCGTGAGCCCAGGTGGCGCTCTAATTCTTAGCTCCTCTCTAAAGTGGGGATC
TAGGAAGAAGTTTCAGAAACTGAGAGACCGTTTGAACATACCAGAGAACTGGATTGAGCTAA
TAAATGAGAATCCAGAGGTGCTCTATCGAGCTCCCAGAACAGGCCCAGAAATATTGTTGCGC
ATTGCAGAGAAAGTCCATAGCCCTGGTGTTGTGTCATCATTGTCTTCTGGCAATGCAGTCTGTA
AAGTCATGGCCTCAGCTGTATACTTCTTATCAGCAACAATTTTTGAAGACACTGGACGCCCTG
AGTTCAACTTCTTAGAGGATTCCAAGTACAGCTTGCTACAAAAGATGGCCGCATATTCTGGCT
TTCATGGTTTCAATGATATGGAGCCAGAAGATATATTATTCCTATTCCCGAACATTGAGGAATT
AGAATCACTGGATTCTATAGTTTACAACAAGGGAGAAATAGACATCATCCCAAGAGTTAATA
TCAGGGATGCAACCCAAACCAGGGTCACTATCTTTAATGAGCAGAAGACCCTCCGAACATCT
CCAGAGAAGTTGGTGTCAGACAAGTGGTTCGGGACTCAGAAGAGTAGGATAGGCAAAACAA
CTTTCCTGGCTGAATGGGAGAAGCTAAAGAAAATTGTGAAGTGGTTGGAAGACACTCCAGAA
GCAACTCTAGCTCACACTCCACTGAATAACCATATTCAGGTTAGGAATTTCTTTGCTAGAATG
GAAAGCAAGCCTAGAACGGTTAGAATAACAGGAGCTCCTGTAAAGAAGAGGTCAGGGGTTA
GCAAGATAGCTATGGTTATCCGTGACAATTTCTCCCGGATGGGCCATCTTAGAGGTGTAGAAG
ACCTCGCTGGCTTCACTCGTAGTGTGTCAGCTGAAATCCTCAAGCACTTTCTGTTCTGCATACT
ACAGGGTCCATACAGTGAGAGCTATAAGCTACAGCTAATCTACAGAGTCCTAAGCTCAGTGT
CAAACGTTGAGATAAAGGAATCGGATGGTAAGACAAAAACCAATTTGATTGGGATCCTTCAG
AGATTTCTAGATGGTGATCACGTTGTCCCTATAATTGAAGAGATGGGAGCCGGAACAGTGGGT
GGATTCATCAAGAGACAACAGTCTAAGGTTGTGCAAAATAAAGTGGTCTATTATGGAGTTGG
GATCTGGAGAGGCTTCATGGATGGATATCAGGTCCATCTTGAGATAGAAAATGACATAGGAC
AGCCCCCAAGGCTTAGGAATGTCACAACTAACTGTCAGAGCAGCCCATGGGATCTGAGTGTC
CCAATAAGGCAGTGGGCAGAAGACATGGGGGTCACAAACAACCAGGATTATTCCTCTAAATC
TAGCAGAGGAGCTAGATATTGGATGCATTCATTTAGGATGCAAGGACCCAGCAAGCCATTTG
GATGCCCAGTTTATATTATTAAGGGTGACATGTCAGATGTTATCAGACTGAGAAAAGAGGAG
GTGGAGATGAAAGTACGGGGCTCTACTCTCAACTTGTACACTAAGCACCATTCTCATCAAGAC
TTACACATTTTATCTTACACTGCATCAGACAATGATCTCAGTCCAGGCATTTTCAAGTCAATAT
CAGATGAGGGAGTAGCTCAAGCCCTGCAGTTATTTGAGAGGGAGCCAAGCAACTGCTGGGTG
AGATGTGAGTCTGTAGCTCCAAAATTCATATCAGCCATCCTTGAGATATGTGAGGGGAAGAG

ACAGATAAAAGGAATCAACAGAACCAGACTCTCAGAGATTGTGAGAATTTGTTCTGAATCTT
CCCTAAGATCAAAGGTCGGATCTATGTTCTCATTTGTCGCCAATGTTGAGGAGGCCCATGATG
TTGATTATGATGCGTTAATGGATCTAATGATAGAAGATGCTAAGAACAATGCATTCAGTCATG
TTGTCGATTGCATAGAGTTGGATGTTAATGGTCCTTACGAGATGGAGTCTTTTGATACATCTGA
TGTCAACCTCTTTGGGCCAGCCCATTACAAGGACATCAGTTCATTATCTATGATTGCTCATCCC
TTAATGGATAAGTTTGTTGATTATGCCATTTCCAAGATGGGGAGAGCCTCAGTTAGAAAAGTT
CTAGAGACAGGTCGGTGCTCTAGCAAAGACTATGATTTATCAAAGGTTCTCTTCAGAACTCTA
CAGAGACCAGAAGAGAGCATTAGGATAGATGATCTGGAGTTATATGAGGAGACAGATGTGG
CGGATGACATGCTAGGCTAAGACCAATAAGCAAAGTCAGGCTTAGATTTAGGGATACTACGC
TAGTATTGGAATCCATGTGGGTTCTGATACTAGCATAGTGCTACAATATTGGGCGGTCTTTGTG
T

>Rift valley fever virus segment M (animal origin)

ACACAAAGACGGTGCATTAAATGTATGTTTTACTAACAATTCTGATCACGGTTCTGGTGTGTG
AGGCGGTTATTAGAGTGTCTCTAAGTTCCACAAGAGAAGAGACCTGCTTTGGTGACTACACCA
ACCCAGAGATGATTGAAGGAGCTTGGGATTCACTCAGAGAGGAGGAGATGCCAGAGGAGCT
CTCCTGTTCCATATCAGGCATAAGGGAGGTCAAAACCTCAAGCCAGGAATTGTATAGGGCATT
AAAAGCCATCATTGCTGCTGATGGCTTGAACAACATCACCTGCCATGGTAAGGATCCTGAGG
ATAAGATTTCTCTCGTAAAGGGTCCTCCTCACAAAAAGCGGGTGGGGATAGTTCGGTGTGAGA
GACGAAGAGACGCTAAGCAAATAGGAAGAGAAACCATGGCAGGGATTGCAATGACAGTCCT
TCCAGCCTTAGCAGTTTTTGCTTTGGCACCTGTTGTTTTTGCTGAAGACCCTCATCTCAGAAAC
AGACCAGGGAAGGGGCACAACTACATTGACGGGATGACTCAGGAGGACGCCACATGCAAAC
CTGTGACATATGCTGGGGCTTGTAGCAGTTTTGATGTCTTGCTCGAAAAGGGAAAATTCCCCCT
CTTCCAGTCGTATGCCCATCACAGAACCCTACTAGAAGCAGTTCACGACACCATCATTGCAAA
GGCTGATCCACCTAGCTGTGACCTTCAGAGTGCTCATGGGAATCCCTGCATGAAGGAGAAACT
CGTGATGAAGACACACTGTCCAAATGACTACCAGTCAGCTCATTACCTCAACAATGACGGGA
AAATGGCTTCAGTCAAGTGCCCTCCTAAATATGAGCTCACTGAGGACTGCAATTTTTGCAGGC
AGATGACAGGTGCTAGCTTGAAGAAGGGGTCTTATCCTCTTCAGGACTTATTTTGTCAGTCAA
GTGAGGATGATGGATCAAAATTAAAAACAAAAATGAAAGGGGTCTGCGAAGTGGGGGTTCA
AGCACTCAAAAAGTGTGATGGCCAACTCAGCACTGCACATGAGGTTGTGCCCTTTGCAGTATT
TAAGAACTCAAAGAAGGTTTATCTTGATAAGCTTGACCTCAAGACTGAGGAAAATCTGTTGCC
AGACTCATTTGTCTGCTTCGAGCATAAGGGACAGTATAAAGGAACAATGGACTCTGGTCAGA
CCAAGAGGGAGCTCAAAAGCTTTGATATCTCTCAGTGCCCCAAGATTGGAGGACATGGTAGC
AAGAAGTGCACTGGGGACGCAGCTTTTTGCTCTGCTTATGAGTGCACTGCTCAATACGCCAAT
GCTTATTGTTCACATGCTAATGGGTCAGGAGTTGTACAGATACAAGTATCCGGGGTCTGGAAG
AAGCCTTTGTGTGTCGGGTATGAGAGGGTGGTTGTGAAGAGAGAACTCTCTGCTAAGCCCATC
CAGAGAGTTGAGCCTTGCACAACTTGTATAACCAAATGTGAGCCTCACGGATTGGTTGTCCGA
TCAACAGGTTTCAAGATATCATCTGCAGTTGCTTGTGCTAGCGGAGTTTGCGTTACAGGATCGC
AGAGCCCTTCTACCGAGATTACACTCAAGTATCCAGGGATATCCCAGTCCTCTGGGGGGGAC
ATAGGGGTTCACATGGCACATGATGATCAGTCAGTTAGCTCCAAAATAGTAGCTCACTGCCCT
CCCCAGGATCCATGCCTAGTGCATGGCTGCATAGTGTGTGCTCATGGCCTGATAAATTACCAG
TGTCACACTGCTCTCAGTGCCTTTGTTGTTGTGTTCGTATTTAGCTCTGTCGCAATAATTTGTTTG
GCCATTCTTTATAAAGTTCTCAAGTGCCTAAAGATTGCCCCAAGGAAAGTTCTGGATCCACTA
ATGTGGATTACTGTTTTCATCAGATGGGTGTATAAGAAGATGGTTGCCAGAGTAGCAGACAAT
ATCAATCAGGTGAACAGGGAAATAGGATGGATGGAAGGAGGCCAGCTGGCTCTAGGGAACC

CTGCCCCTATTCCTCGTCATGCTCCAATTCCACGTTATAGCACATACCTAATGCTACTATTGAT
TGTCTCATATGCATCAGCATGTTCAGAACTGATTCAGGCAAGCTCCAGAATCACCACTTGCTC
CACAGAAGGTGTCAACACCAAGTGTAGGCTGTCTGGCACAGCATTAATCAGGGCAGGGTCAG
TTGGGGCAGAGGCTTGTTTGATGTTAAAGGGGGTCAAGGAAGACCAAACCAAGTTTTTGAAG
ATAAAAACTGTCTCAAGTGAGCTATCGTGCAGGGAGGGCCAGAGCTATTGGACTGGGTCCTTT
AGCCCTAAATGTCTGAGCTCAAGGAGATGCCATCTTGTCGGGGAATGTCATGTGAATAGGTGT
CTGTCTTGGAGAGACAATGAAACCTCAGCAGAATTTTCATTTGTTGGGGAAAGCACGACCATG
CGGGAGAACAAGTGTTTTGAGCAGTGTGGAGGATGGGGATGTGGGTGTTTCAATGTGAACCC
ATCTTGCTTATTTGTGCACACGTATCTGCAGTCAGTCAGAAAAGAGGCCCTTAGAGTTTTCAAC
TGTATCGATTGGGTGCATAAACTCACTCTAGAGATTACTGACTTTGATGGCTCTGTTTCAACAA
TAGACCTGGGAGCATCATCTAGCCGTTTCACAAACTGGGGTTCAGTTAGCCTCTCACTGGACG
CAGAGGGCATTTCAGGCTCAAACAGCTTTTCCTTCATTGAGAGCCCAGGCAAAGGGTATGCA
ATTGTTGATGAGCCATTCTCAGAAATTCCTCGGCAAGGGTTCTTGGGGGAGATCAGGTGCAAT
TCAGAATCTTCAGTCCTGAGTGCTCATGAATCATGCCTTAGGGCACCAAATCTTATTTCATACA
AGCCCATGATAGATCAGTTGGAGTGCACAACAAATCTGATTGATCCCTTTGTTGTCTTTGAGA
GGGGCTCTCTGCCACAGACAAGGAATGACAAAACCTTTGCAGCTTCAAAAGGAAATAGGGGT
GTTCAAGCTTTCTCTAAGGGCTCTGTACAGGCTGATCTAACACTGATGTTTGACAATTTTGAGG
TGGACTTTGTGGGAGCAGCCGTGTCTTGTGATGCCGCCTTCTTAAATTTGACAGGTTGCTATTC
CTGCAATGCAGGGGCCAGAGTCTGCCTGTCTATCACATCCACAGGAACTGGAACTCTCTCTGC
CCACAATAAAGATGGATCTCTGCATATAGTTCTTCCATCAGAGAATGGAACAAAAGATCAGT
GTCAGATACTACACTTCACTGTACCTGAGGTAGAGGAGGAGTTTATGTACTCTTGTGATGGAG
ATGAGCGGCCTCTGTTGGTGAAGGGAACCCTGATAGCTATTGATCCATTTGATGATAGGCGAG
AAGCAGGGGGGNAATCAACAGTTGTGAATCCAAAATCTGGATCTTGGAATTTCTTTGACTGGT
TTTCTGGACTCATGAGTTGGTTTGGAGGGCCTCTTAAGACTATACTCCTCATTTGCCTGTATGTA
GCATTATCAATTGGGCTCTTTTTCCTTCTTATATATCTTGGAAGAACAGGCCTCTCTAAAATGT
GGCTTGCTGCCACCAAGAAAGCCTCATAGATCAGTACGTGTAGAAGCAATATATAGAAATAA
GTAAACATAAGCAAATCTAATTATGTAAATATTGTACAGATGGGTCAAACTATTGGGATATCC
AAGTTTAGAATCTTGTACAATAGTACTTTAGATGTAAGCTTAGTTGTAATTTGGGGTGGTGGGG
TGAGGCAGCAGTAGTCTCAAGTACATGTGGATATTCTAGTTAATGTGAATGTCTTTTGCCAGAT
TAGCTGGGAATTAAACTAACTCTTTGAAGTTGCACCGGTCTTTGTGT

>Rift valley fever virus segment S (animal origin)

TCCGTACAGTTATACGTATTACTAAAGGTTAACCTGGTAACTGGGACACAAGACTCCAGCACA
GGACCACCTGAGCCACCTCCAACCACCACCAGCAAAACCAGCAAAAACCTTTTCAGAAACAT
ATTGATCCTAATAAGCTAGGCCATATGACTCACATTCAGTGTGTTCGGCTCCTGCAGGGGCAG
CAAGAGAGGATATTTCTAATGCTGTAGTTCCAAACTCAGCTCTCATTGATCTTATGGAGGGAG
AAGAAACGATGCTGCGCTCATCCACTCCCCTAGCATGATGGGGAGAAACAACTGGAGTTCCA
GTTGTTCCTCCAATCCCAGATGTTGAGATAGAATCAGAGGAAGAGGTGATGACGATGGATATT
GTTGAGGTTAACCTGGTAACTGGGACATAAGACTCCAGCACCACCACCCCACCCCCAATCCC
GACCGTAACCCCAACCACCCCTTTTCCCCAAACCCCTGGGCAGCCACTTAGGCTGCTGTCTTG
TACGCCTGAGCAGCTGCCATGACAGCTGCTGACGGCTTCCCATTGGAATCCACAAGCCCAAA
AGCTTTCAAGAATTCTCTCCTCTTCTCATGGCTTATAAAGTTGCTATTCACTGCTGCATTCATTG
GCTGCGTGAACGTTGCGGCAACCTCCTCCTTTGTTCTACCTCGGAGGTTTGGGTTGATGACCCG
GGAGAACTGCAGCAGATACAGAGAGTGAGCATCCAATATTGCCCTTAGATAGTCTTCTGGTA
GAGAAGGGTCCACCATGCCAGCAAAGCTGGGGTGCATCATATGCCTTGGGTATGCAGGGGAT

AGGCCATCCATGGTGGTCCCAGTGACAGGAAGCCACTCACTCAAGACGACCAAAGCCTGGCA
AGTCCAGCCAGCCAGGGCAGCAGCAACTCGTGATAGAGTCAACTCATCCCGGGAAGGATTCC
CCTCCTTTAGCTTATACTTGTTGATGAGAGCCTCCACAGTTGCTTTGCCTTCTTTCGACATTTTC
ATCATCATCCTCCGGGGCTTGTTGCCACGAGTCAGAGCCAGAACAATCATTTTCTTGGCATCCT
TCTCCCAGTCAGCCCCACCATACTGCTTTGAGAGTTCGATAACCCTACGGGCATCAAATCCTT
GATAAGCAAACTCTCGGACCCACTGTTCAATCTCATTGCGGTCCACTGCTTGAGCAGCAAACT
GGATCGCAAGCTCTTGATTAGTTGTCCATTATTGTAATGTAGTGTTTGTATCTCTAAAAGGGAG
CNNNGTGG

> Dugbe virus segment S (animal origin)

TCCCACAGTAACACCACNNGCCGGAGCTCTGCAGATCTCGCCCGGTGTGCTTACTTTGAAAG
AAGCAAGATGGAGAATCAGATCAAGGCAAACAATNAAAAAGAGTTTGATGAGTGGTTTAAA
CCTTTTAGTGAAAAGCTCCAATTAAGGTCAAACCTCACAAACTCTGCATCCCTGTGTGATAGA
GTACCTGACCTAGCTCTAGCAGAAATGAAAATGGCCCTTGCCACAGATGATAAAGAAAAAGA
CTCAATCTTTTCTAATGCTCTGGTGGAGGCAACACGTTTCTGTGCTCCAATTTATGAGTGTGCTT
GGACCTGCAGTACAGGCGTAGTGCAGAAGAGCTTAAGCTGGTTTGATAAGAACAAAGACTTC
ATCAAGCTCTGGGACGCAAAGTACATGGATCTCAGAAAGGGTATCCCTGAACCAGAGCAACT
AGTTTCATACCAGCAGGCGGCCCAAAAGTGGAGAAAGGACGTTGGTTATGAAATTAACCAAT
TCACAAGGTCTCTCACACATCCAGTTGTTGCAGAGTACAAAGTGCCTGGAGAAATTGCTGTTG
ATGTCAAAGAAATGCTGTCTGACATGATCAGGCGCAGGAATGTCCTGCTGAACGGAGACGGA
GAAAATGCTGGAAAAAAGGTCCTATCAGCAGAGAGCATGTCTCTTGGGGCCGTGAGTTGGC
AGGAGGAAAGTTCCAAGTTGTGTTTAATCCTCCTTGGGGGGATATAAACAAGTGTGGTAAATC
AGGCATTCCTCTAGCAGCTACTGCCATGGTGAAGGTTGCTGAGCTAGATGGGTCAAAGAAGC
TTGAAGATATAAGGCAGGCCCTTCTAGATCTAAAGAAATGGGTTGAAGATAACAAGGATGCA
CTAGAGGATGGGAAAGGCAATGAGCTCGTCCAGACTATGACAAAGCATCTGGCTCAAGCAGT
GGAACTATCCAAAAAGTCCAATGCCCTGCGTGCACAAGGAGCACAAATAGACACTCCTTTCT
CAGCCTTTTATTGGGCTTGGAGCGCTGGGGTTAAGCCAGAGACATTTTTCACTTTGTCTCAATT
CCTCTTTGAGATGGGCCAGAATGCTAGAGGAGGCAAAAAAATGATCAAAGCACTGACTTCCA
CACCACTAAGGTGGGGCAAGGGATTGATTAACCTGTTTGCAGATGATGACTTTTTGGGCAACA
GACTATACATGCATCCTGCAGTTCTGACTCCTGGTCGAATGAGTGAAATGGGTGCATGCTTTG
GGGTTATTCCTGTTGCAAGCCCAGAAGATGCAATCCTAGGTTCAGGACACTCAAAAAACATA
CTGAACTTCAAAATTGACACCAGTGTGCAGAACCCATGTGCATCCACAATCGTGCAACTATAC
AGAATCCAGAAATCTGGCTTTGATCTGGAAAGCTTAGAAGTAGTGTCTACTGAACATCTTTTG
CACCAGTCATTTGTTGGTAAAAGATGCCCAACCCAGAATGCTTACAAGGTGAGAGGAAATGC
AACCAATGTTAACATCATCTAAAAGCTGGCATTGTGTGGCCTTCCCTAACATCTTAAACAAGT
TGCACATCAGCGAAAGGCCACTAAAAGCTTTTTTCTTGCTTATCTTTCCATTTTTCCTTCTTTAT
ATATTAGTTTTGCTTTTTTGACTAACATACTAACATAGTATAAGTTGTAATCAATCTGCTAATTC
TGCTTAACACAGGGGGGCTGTGCGGCAACGATATCTGCAGA

> Dugbe virus segment M (animal origin)

TCTCnaAGAcATacCTGCGGCACATACAAGTGTTAAAGGTGTTCACAATGTCTAAGAGGGTACT
GATCATAGCCGTAGTGGTTTGTCTGGTATTCACCACTCAAAACCAGATAGCCGGCAACCACAC
AACAGCCAACAGCGGCAGCCCGTCCACAAAAGAGGCAAGCAGCATGCCAACAGTCAGCGAC
ACTCCACAAACAACCACCACCTCCACTGCAGTGTCCACCACCATCACCGCAACCACCACTCC

AACAGCTTCATGGACCACACAGAGTCAATATTTTAATAAAACAACACAACACCATTGGAGAG
AAGAAACCATGGTCTCAAGGAATCCTACTGTGCTGAACAGACAGAGAAGAGCAAGTTCTGTG
AAAGAACTACTCAACACCAAGTTCTTAATGTTGTTAGGTTTCATTCCAAAGGGTGAAGTCAAT
CACCTAGAAAATGCCTGCAACAGGGAAGGGAAAAACTGTACAGAATTGATACTGAAAGAAA
GGATAGCTCGGTTCTTTTCTGAGACAGAAAAAGAGTCATGTTATAACACTTACCTAGAAAAAC
ACCTTTGCAGTGTTAGCCCTGAAGTTAGTCTCACCcCTtACAGGGTCCTCGaGCTAaGAGAAGA
CATTTTGCTAAAGAAAATCGACCGTAGAATCATCAGATtTgcaGCAGaCTccCaaAGaGTGACCTG
TcTcTcAGcATctcTACTAAAACCtGAtGTCTtTATTAgGgAACAGAGAATTGATGCTAAGCCcTCCA
ACGGTCCTaAAaTAGtGCCTGTGGACaGCGTGCGCTGcATAAACTTAgAAgCTAACGtAGATGTGa
GATCAAACAAATTAGTGATACAGtCtTTGATGACcACAGTTAAAATTAGCCTGAAAAaCTGTAA
GGTCGTAGTCAATTCTAGACAGTGCATCCaTCAGcaaACagGTTCTGGTGTTAtcAAAGTCCCAAA
aTTCGAAcAgCAGCAgggTGGGACTTGgtCaAGctAcATCGCCGGGGTGTACACAGCAACCATTGA
CCTGTTAGATGAAAAAAaCCAAAACTGCAAGCTATTTACAGAATGTATTGTCAAAGGTAGAG
AGTTGGTTAAAGGACAAAGTGAACTTAAGTCCTTCAATATAGAAGTCCTCCTACCGAGAGTG
ATGAAGACTAGAAGAAAGCTGTTAGCTGTCACTGATGGTAGCACTGAATGTAATTCAGGGAC
ACAGCTAATTGAAGGAAAGTCCATAGAGGTCCACAAACAAGACATAGGAGGGCCAGGGAAA
AAGCTGACCATTTGTAACGGCACTAGTGTTCTCGATGTACCCCTGGATGAAGGACATGGATGC
TACACCATTAATGTAATCACGTCCAAAAGGGCATGCAGACCAAAGAACAGCAAGTTACAGTG
TTCAATTGACAAAGAGCTTAAGCCTTGTGACTCAGGCAAGTGTTTGAGTATCAGCCAAAAaGG
AGCAGGCCACATTAAGGTGAGCAGAGGGAAAACCATTCTTATTACCGAATGCAAAGAACACT
GCCAGATCCCAGTACCAACTGGAAAAGGGGATATAATGGTGGACTGCTCAGGAGGCCGTCAG
CACTATTTGGAGGTGAACATCGTTGATATCCACTGCCCTAACACAAAATTCTTAGGAGGAATA
ATGCTTTATTTCTGTAGAATGTCAAGCAGACCTACAGTAGCAtTGCTtCTtGGCATCTGGATTgG
CTGCGGCTATATACTCACATGCATCTTCTCATTTTTGCTATACCACTTAATTTTATTCTTTGCAA
ACTGTATAAAACAGTGCAGAAAGAAAGGAGAGCGGCTTGGAGAAATCTGTGTCAAGTGCGA
GCAGCAAACAGTTAACCTTATGGACCAAGAGCTGCATGACCTCAATTGCAATTTCAATCTTTG
TCCTTATTGCTGCAATCGAATGTCAGACGAAGGTATGAGCAGGCATGTAGGTAAGTGTCCTAA
AAGATTAGAAAGACTTAATGAAATAGAGCTCTATCTAAACTACAAGCGAGTGCCTTCTTGCCT
AAGGTGTATGCTATCAACTTCTATAAGCGTAGGCATTTTCTTGAAAAGAACAACTTGGCTGGT
TGTACTTCTGGTTCTGTTAGGATTAGCCATTTCACCAGTTCAAGGAGCTCCaGCAGAGGTCAGT
AATGTTAAACAGGACGGTGACTACAGTATCTGCTACTTTATTTTTGGTTGTTTGGTGACAGCTG
CACTTTTGTTGAAGGTCAAGAGAACAAACTCTAATGGCATTGTGGTAGTGGTCGATAGCTTTG
GAAGATGTCCATACTGTAATGAATTCACTGATTCATTATTTGAGGAAGTGCTTCACGATACAC
TATGCAGTCTCTGTGTCTGTCCTTTCTGTGAGAAACAGGCTCTTGATCTGGTGACCCTTGAAGA
ACATGTCAAAGAATGCTACAAAGTTACAACTAGGAAGGACATATTCAAGATATTAGGGAGAA
AGTTCACCAATGCTCTAGTTAGAAGGGAAAAACTGTTCACAACAGGACTGCAGCTCTTCATA
AATAAAACAAATGTTGTCGTCTTTGCTCTCATTATGTGCTTCTTGCTACTGTTAACAGGACATA
ACGCAAGTGCTTTTGATTCTGGTGACTTGCCAGATGGGATATGGGAAGAATCAAGCCAACTTG
TTAAGTCATGTACACAGTTTTGTTACATAGAAGAAGATATCTGCtAgaGCtCCTGCTGaGaaTGgC
GTgGGAATGAAtTcCTgTTCTTcAAtgCCTTcAGcAATTcAcTGAAAAGACTTTCAGATTCTCACAA
GTTGTTGACAAGTGTCTCAATTGATGCACCTTGGGGTGCAATTAATGTTGAATCAACCTGGAA
ACCAACACTGGCAGCATCCAACATTGCCATGAGCTGGAGCTCCACTGACATCAAAGGTGAGA
AGGTTATACTATCAGGAAGGAGCACATCCATAATAAAGCTTAAAGAAAAAACTGGTGTGATG
TGGGAGCTTGGGTCAGAACTAGCATCAGAAAAAAaGAAACTTTTAGTTTCGATAATGGATTTT
GCCCAAGTCTACAATTCAGTATTCCAGTACATAACAGGTGATAGACTACTAAGTGAATGGCC
AAAAGCCGTCTGCACAGGAGATTGTCCTCATAGATGTGGCTGCCAAACATCAACTTGTATGGC

CAAAGAGTGGCCACACACAAGAAACTGGCGGTGCAACCCCACATGGTGTTGGGGAATTGGTA
CTGGTTGCACCTGCTGTGGTATGGATGTGGAAAGGCCATTTAATAAATATCTTGGTGTTAAGTG
GTCAACAGAATATCTTAGAACTGAGGTGTTGGTCTGCGTGGAGGTCACGGAGGAGGAGAGGC
ACTGTGAAATTGTCGAGGCAGGAACCAGATTCAACATTGGCCCTATAACAATAACGATTTCC
GATCCCCAAAACATTGGAAGTAAGCTTCCAGAAAGTTTAATGACAGTCCAAGAAATTGATGA
CAGCAACTTTGTCGACATAATGCATGTTGGTAATGTTATCTCTGCTGACAACAGTTGTAGGCTT
CAAAGCTGCACTCATGGCAGTGCCGGTGACTACCAGATTTACAGCACTGACAGCTTAATCAA
AGATGATCACAGTTCTGGACTCAACCTAGCTGTGTTAGATCCCAAAGTCAACAGTAGCTGGCT
ATCATGGGAAGGGTGTGACATGGACTACTACTGTAATGTTGGCGATTGGCCAACCTGTACCTA
CACTGGTGTAGTGACTCAAAACTCAGAGAGTTTCTCAAACCTGATCAACATAGAAAAAGATT
ACACACAACGCTTTCATTTTCACTCaAAAAGAATCTCAGCAAAAGGTCACACACTTCAGTTAG
ACTTAAAGGCAAGACCTAACCAGGATGGAGGTGAAGTGACAGCACTGATTGAAGTTGATGGT
ATGGAGTTACATTCAAAGACAATAAGGCTAAGTGGTATAAGACTAACTGGACTGAAATGCAG
TGGTTGTTTTAGTTGCACTTCTGGCATATCATGCAGCGTCAATGCCAAGTTGACTTCTCCAGAC
GAATTTACACTGCATCTGAGGAGCACCAGTCCCAATGTGGTAGTGGCAGAAACTAGCATAAT
TGCAAGAAAGGGTCCAAGTGCCACAACTAGCAAGTTTAAAGTGTTCTCTGTTCGTGATACAAA
GAAAATTTGCTTTGAGGTGGTGGAAAGAGAATACTGCAAAGATTGTTCTCCTGATGAACTCAC
TACATGCACTGGTGTTGAGTTAGAACCTCCCAAAGACATATTGTTAGAACATAGAGGAACAA
TAGTACAGCACCAAAACGACACTTGCAAATCTAAGATTGACTGTTGGTCAAATTCTATAAGTT
CATTCGCATCTGGTATAGGAGATTTTTTAAGCATTACATTGGAAGCATTGCTGTCGGAATACT
TGGCACGGTTCTTCCTTTCGCCCTTTTAATTCTTTTTTTCATATACGGTGACAAAATGCTTTGGC
CTTTCAAAGTTTTTTGTAGGCCATGTAGAAGGTGCTGTAGAAAAAATGAAGGATACAACAAA
CTGGCTGAAGAAGAAGAGCTGAGAGACATAATAAGAAAGTTCAGTAAAAGTGGTGAACTTA
TCAACAAAGATGCAAAAGATAAGAGAACTCTTGCAAGACTATTCATGTCTGACAACCCAAAA
TTAAAAAAAGAAAAAAAACTTTCTGAAATTGCTTAACAGGTACTAAAACTAGGTGTACTCAC
CACAACCACCCAAATCAAGAAGACCTAGTGAATTTTATGTAGTTCTAACTTGCTCACAGTTTA
AGGTTCTTTTAACTTACTTACTCTATTAATCCACTATGCCTGCTTTTGCTAACTCTAATTTTAAA
ACTTGCTGTGCCGCCACGATATCTGCAGA

> Dugbe virus segment L (animal origin)

TCTCAAAGATATCAATCCACCTTTTCCCCAAAACTTNAACATGGACTTCTTAGACAGTCTAATT
TGGGAAAGGGTTGTTGATGAACAATATATCACTAACCCAACATTTTGTGTTAGTGACTATTTTG
AAGTTATCAGACAGCCAGGGGATGGCAACTGTTTTTACCATAGCATTGCAGAACTGTTCTTTG
ATGTCAAAACACCATCTAGCTTTAGAAAAGTAAAGGAGCATTTACAGTTAGCAGCAGAAGTG
TACTATGACACTGAGCCAGAGGCAGTAGGAACTGGAATCTCAAAAGATGAGTACATCAAGGT
CGCAATGAAAGACAATGAATGGGGAGGTTCCTTGGAAGCTTCAATGTTATCAAAGCATCTCC
AAACAACCATCATCTTGTGGGTGGTCAATTCAACAGAACAGGTTACGGCCGCCATTAAGTTTG
GACCAGGTAGGGTGTCAACAGCTCTAAATCTTATGCATGTTGGTCGCACACACTTCGATGCGC
TTAGGATAATAGAACAGCTGGAGAACAACCAACCTCAAGACAGGAATAGGCTAGACATAGC
AGACAGAATTGCAGCTGCGGAGGTTTATGTGAGCCAGTCTATAGAGGACAATTTGCAAGAGG
ACGAATTCTTTGACTACGCAAGAGAGGATGAGATTTCAGAAGATGTCAGTGCACCAGGAGGC
TCCAGAGAAGCAACTGAACTAAAAAAGAAGGCAATACTTTTGAACAAAACTGTTAAAAGAG
GAGAAAACATACCAATAAGGGTAGGCAGAGTCCTTGACTGTCTATTCAGCTGCAAGATTGCA
GTTAGTTTAGATGAAGGGCTACTCTATTTGAGACCTGAAACCAGGGAAAGTGAAGCCACCTC
AATTTCTCTAAGACAACTTGGACACAAGCTACTCACCCGTGATAGGCACATTAAAATGGAAT

ATGCTAGATCCAAACTCTATGTGACCAGGGATCTCATAGATCACCTGGATATAGGTGGCTTGC
TTAGATCATCATTTCCTGGCTTAGGATTAGAGAGGTACATACAGCTGTTGCACTCAGAGCTTGT
GTTAGACTTGGTAACAGTAGTGCTTGCTGTACTATTATCAACTTTCTTGTATGGCTCAAACAAC
AAGAATAAAAAACAGTTTATAACAAACTGTTTATTAAATACTAAGCTTTCAGGCAAAAGAGT
GTTTAAAGCATTATCAAAATTAACTGGTCAAATGTTGTATCGTACTCCAAAAAGGGCAGTATC
AATTGTGTCTCAAGAGCTCTATGGGAAACTAATGCTAAAGGTGAAGAACAACTTAGAGGGAA
TGGGACCAATAAGCATGCTAGCATTAAGGAATCTAGACTTTGACAATATGCAGCTTCAAGACT
ATCTGGAAATGTTATCTGAAATGTCCAAGATTGACAATTCTGATGTTGAATATACTCACAGAG
AAATAAGTGACCTTCATACATTAGTTGAAAGGCTAAGTAAACTTCAGAAGTCTCAAGATGTTA
ACGAACTCAAGCTATGGTTTAAGGAAGAGGTGTTGACAAAAAGGTCACAAAGGAGTGTTGGC
AATGCCTTTGAGTTTTTAATCAATGATTACTTTAAGAAGAAGGACATTATGAAGTTTGTTTCTA
CATCAGGGAAAGCATCAAGTACTGGAAACATTGGAAATGTGCTATCATATGCACACAACCTG
TACCTCAGTAAGGAAAGTCTTAGAATGACCTCTGAAGATGTTACTCAATTACTAATAGAAATC
AGAAAGTTGCATAAACTACAAGGTGATCTTAGTATTGAGCCAGTAGCCATAATTTGCGACAA
GCTAGAGGACCAATTCAGAAAGCTATTCAGGGAGCTGCCTGAAGAATGCAGCAGCGAATGCC
AGACACTATTTAATGACATACGCAACTCTCCAAGCCACAGTGTTGCGTGGAAACATGCTCTTA
GACTGAAAGGAACAGCATATGAGGGGATGTTTGCAAAACAATATGGTTGGTCGTACATCTCA
GAAGATATTAAACCTAGTTTAACAATGATTGTTCAAACTCTTTTCCCAGAGTCCTTTGAGGCAT
TTTTGGATAGGACTCAGCTACATCCAGAGTTTCGGGATCTCACCCCTGATTATGCTCTAACTCA
GAAAATATTCTTTCCCAGAAACACAATACCTAGAACAGAAAACAGACAGCTTGCAATAGATG
TCTCACTGGAAGGCTCTGTTGAGGCTGTACCCGTTGTGGAAAAAAGAATGTTTCCTCTTCCTGA
GGTTCCAATTGGAGAGGCTAACTCAATCAGTAGAGTTATGAATATATTCAAGGAGAAAAGGA
AAGAGAGCATGCAAAAGAAACTTGAGCATGATAGACAGGCAGAAGCGAATCGACTAAAAA
GTGCTGGATTAGGTGCCTCCAAGGCAGAGCAGGAAGTTTGCAACAGCGCTCAAGACAGAAA
AGAGGAAAAAGAGAGAACCATAGAGCCTGCTGGAAAACAGCAGAGAACAGAAGACCTTGT
TGTCATTGAGGGCAGCCAAGATGAAGGTAATAGTGATCCACAGAAGAAAGTCGATGAAAAG
ACAGTTCCGGGGGAGTCAAAACAACACTCCAAAAGCAGTAAATCAAGTAGCACGAACCAAA
TGAGCCAAAAGGTTGTAGATGTTCCCTCAGTAGAAGATAGCTCCGATCAAGCACCTGGTGATT
TTCCAGATTATGGATACTACTTTAAAAGAATTGTCATGGATGAGAATGGCACAGTGCTGACTG
AAGAGGCACAACTAGAAAAGAGACAGTTACTCTTTATTGAAGTAGGCTACCAGACAGATGTT
GACGGCAAAATTACTACAGATTACAAGAAGTGGAAGGACATTCTGAGGTTGCTAGAGCTTCT
AAACATTAAATGCTCCTTTATTGCATGTGCAGACTGTTCTTCAACACCATCGAACAACTGGTG
GATTAGTGAAGATAAAGTCAGGCTTTTAAAAAATTCAATTAGTCATCTGTTTTCAAAACTGAC
CCAGAATTCTCCTGCAGATATCACAGACATAGTAGTTGGTTCCATAAGCACACAAAAAGTTA
GAAGCTATTTAAAGTCTGGTACAGCAACCAAGACACCCATTTCACTGAAGGATGTGCAGGAA
ACATGGTCTAAAATGAAAGACTACATAGTAAACAGGCCAACTGGAATTTCACTGAACAAAGA
GCTGGTTGGTGCCCTTTATCAAGGTCTTGTGGAAGGAGCAATAATTAGTAAAGAAGGCACAG
CCAACTTAATACAGATGTTAAAAGACAAGCAGGAAAGAATCACAGATGAGTTTGAAAGAAC
AAAGTTAAAACATGAAGTCAATGAAGATGTCAAGACAAGTGAGAAATTACTGTTAGGTTGGT
TAATGGAAGACCTCAAAGGCTGCCGTTGCATGGGATGTTTAACTAAAATAAAAGAACTCTCT
GAATCAATGTCAGTAAACCAGGATAGGCTGGAATACTTGTCAACCAACTGCCAAACAAAGAG
TCACTGCACTGAATGCCACCCAAGAAGCTTAGAGTATAGAAATATTTCAAATGTGGACAATA
GGGTTCCCAGCATGCAGAGAGTCAGTCATTCAAGAAATGAAGGTTTTGAAGACACAAATGAG
ACACTCACTGAACTAGATAGGCTTGTAAGATTAACACTGCCAGGTAAGACAGAGAAAGAGA
GAAGAGTGAAACGTAATGTTGAAGGTCTAATCAGGTTCATGATGCAGCAGTCAAGCTTAGAC
TGTATAAAGCTACCATCTGGACAAATAATTGCTCACAGATGCAATAAAAAATTTAAAAACAG

TTCAGAAGCTGAAGAGAAGTGCAACGAAAGATTTGAGAGGTTAATGAAAGAATTATCAGAG
CAGAAGCTTAAGCCCTACTCAGATCATGTAAAGAAGACAATCACAAGTTCCTTAAAAAAAAC
AGACAAGCAAGCAGGTTCTAAGTGTGCTGTACCAAGGTTGTGGCTAGAAACACTAATCAGAG
ACTTGAGGGTTCCTACAAAGGATGAGGAGATTCTTCTCAACATACGCACAAGTATGCAAAGT
AAGACCAACTTCATCAGAAATAATGATAAACTCATTATCAGAAGTAATAAGGAAATTGCTGA
TTACTTAGAAACTAAAAGGAAAAATTTGTTGTCAGAAAAGGCCTCTGATAAAATCTTTTCATC
TGATTGTATACTTTTCAAAGAGGTTATTGCGGAAGCTCTCAGGAGGTACTACAGTACTCCCTAT
GAAGGTGTGCCAGAAACAATAGTAAAACTCATCAACTTTTTGTGCACATTTGGTTGGTTTCAG
GAGGTAGTCTTATACAGCAAGATCTGTGAAACTTTTTTGAGATGTTGTACAGAGTTTAGTAGAT
CAGGAATTAAACTGGTAAAGGTGAGACACTGCGACACCAACTTATCAATAAAACTGCCATCA
AATAAAAAAGAAAACATGCTCTGCTGCCTTTATGATAAGAACATGTCCCTCTTAAAAGGCCCT
TTTTTCCTAAACAGAAGACAAGCAATTCTCGGCAGTGCCTATCCTTACATACTAATAACTTTGT
ACATTCAAGTTCTGCAGCAACACAGATGTCTAGAAGTGTTAAACAGTGTTAATGACAGAGTTG
TTGGCAATATTAACACCTGCACCAGCAATCTTCTGAACACAGTTAAAGCAGAACTAACACTG
GTAAACTCTGGACTTTTTGAAAAAGCATATGAGTGTAGAACAGAGCAGTGTAGGTTAGGAGG
CAACTTTTTGAACAGAAGTAGTAGAGACCATTTCATTTCAACTGTATCAGGCCTTAATGTTGTC
TACGGTGCATTGATTAAAGACAATCTGCTAGCTAATTCTCAGCCGCAGAACAAGCAATTACA
GATGCTCAGATTTGGCATGTTATGCGGGCTAAGCAGATTGTCCAGCGCTCTAGAGCTAGGTAA
GAAGTTCTCTACAAGCTGCAGAAGGATTGAGGATAACATTATGAGACTGTATTTGCAGTCAAC
AATCTACTCTGCAAACAGAGATGTAAGTCAGAATGTGCAAAACTGGAAGGTGAAAGATTTAT
GCCCAGATATCACTATCCCTTGTTTCTCAGTTTATGGTCTCTTTGTTAACAGTGACCGGCAGCTT
ATATTCGACATTTACAATGTACATATTTACAACAAAGAGATGGACAACTTTGACGAGGGGTGC
ATTACAGTCTTAGAGGAGACAGCTGAAAGGCATATGCTTTGGGAACTTGACCTTCTAAGATCT
TTGGAGGGAGACACTAGGGATGTTCGAGCAGCAAGGCTCCTGCTAGGTTGTCCCAATATAAG
GAAGGCAACTGACAAAGATGGAAACAGGCTAATGAAAAAGGGAATTACAGATGACTTGAGG
GAAGAAGCTGGTAGTGACTCCTCTAGTATTAGTGGCAGAAGGTCATATGCCTCGAGTGGAAC
CAGAGTGAAAAGCATGTTCGGCAAATACAATTCTTCTCAAAAACCATTTGAGCTTAAGCCTGG
CCTAGAAGTGGTAAATGACCCTCTTCATGATTATAAGCAGGCTGTACAAGATAGCTTCTGTTA
CTCAGAATACACCCCTAACACAGAAAGTGTGTTAAAGGACTGCATTCATATAATAAGAACAA
ATCCAAGTCACACAATGGGTTCATATGAGTTAATTCAAGCAGTGACGGAAAATGCGAGACGA
AAATATCCTCCAGAAAATATAGAAAGAGCAAGAAAGGATCCAAAAAACTGGGTCAGCATAT
CTGAGGTCACTGAAACAACAAGCATCGTCTCACAACCTAGAACACATTTTATGCTCAAAGAC
TGCTATAAAGTTCTACTAGGAACAGAGAACAAAAAAATTGTGAAGATGTTGAGAGGTAAGCT
CAAGAAACTAGGTGCAATGAGGACAGATATTGAGATAGGAAAGAAGGATTGTCTAGATCTGT
TGACAACTGTTGATGGTCTGTCTGAGGAACAGTGTAAGAATATAGTAAATGGTATATTTGAGC
CTTCTAAACTATCTTTCTATCACTGGAAGGATCTTCTAAAGAAAGAATTAAGTGAGGTTTTACT
AACTGATGATGGTAACTACATTTATTGCTGGCTCAAAACATTATCTTCAATGGTCAAACACTC
GTTGAAAAAAGACTTGCGGTTCATGACAGGTAAAAATTCCTTGGATATAAAGCCAGAAATGT
TCACAGACGAAGAATATTCAGCTCTGAACATTATGAAGTTAGAATTGTTGGGCGAACAAGCC
GATGGAATACAGGGCAAAACTGACTTCCTCTTGAGTTCTTGGAAAAAATGTGCCCTAAAGCC
CAAAGAAGGCCAGTCAATATTAAATGTAGGGCTAAATAGTCTGGCAGCACTACATGATGAGC
TCTATGATATTAGATTGCAGCATCTTGAGCTAACCCGAATAAAGAAAGAGAACCCAACTGTT
AGCTTCACAAAAGAAGAAATTTTAGTCAAGCGGCTTGAAAAAGGATTCTTAAACAAGTATAA
GAAAGAGGTGATGGAGGCAGTCAATTTAATCTTTTATTGCTGCTTAACAGCGCCTTGGTGTCT
GCACTACAAATCACTAGAGGCATACCTTGTCCGACACCCAGAGATACTTGAAACAGAGTGCA
TCAAAGAAAATGACATACCTCTTCTAGACTTAACTGTCACCAGTCTAATTAGGAGCCTCATTG

ATGATATTGAAGGTGAGTCAAGCTTTAATGATTCTTCAGATATTAAAGTCAGCTTTGCAGTTAA
GTATTTGATAACCCTGTTTACTGCAAATGGAGAGCCATTTTCCCTCAGTTTAAATGATGGTGGA
CTCAGTGATGATCTTCAGTTGACAACTGATGAGAAGCTACTATATCAAACAAAAAAAGTTTTT
GCTAAACTTGGCCTCTCAGGAAATAATTATGATTTTATTTGGACTTTGCAGATGATTGCCAACA
GCAATTTTAACGTGTGTAAAAGGCTTACAGGCAGAACAACTGGGGAGAGGTTGCCAAGAAGT
GTAAGGAGCAAAGTCATCTACGAAATGGTTAAGCTAGTAGGAGAAACTGGAATGGCAATACT
ACAGCAGTTAGCATTTTCTCAAGCCCTGAACTACGACCATAGATTTTATGCAGTCTTGGCACC
CAAAGCACAACTTGGTGGCTCTCGAGACTTGCTAGTTCAGGAAACAGGCACTAAAGTAATTC
ATGCCACAACAGAGATGTTTAGCAGGAATTTGCTTAAGACGACATCTGACGATGGCCTTACA
AACCCACATCTAAAAGAAACAATATTAAACATTGGACTTGACATGTTAAGTACAGCCAGAGC
ACTGGACGGGAAGCAGGTTTCATGACGTATTGCAANNGGTTAAATTTCTTTAAGGCAGCTCTG
CATATCTGGTGATAACACCAAGTGGGGCCCAATCCATTGCTGTTCTTTTTTTTCAGGTATGATG
CAGCAACTACTCAAGGATGTTCAAGACTGGAGTTCGTTCTACAAGTTGACATTTATCAAAAAC
TTATGCAGACAAATTGAGATCCCTGCACCTAGCATTAGGAAAATACTGAATGTACTTAGATTC
AAGCTGAGTGACAAGGGAGGTGTAGAAAAGCTTTCAGAAGAGGCCATAAGGTCAGAATTAA
TCAACAATCTAGCAGAATGGGAAGGGAACGATACTGTTAAATTTTTAATAACAACCTACATC
AGTAAAGGCATTATGGCAATGAATAGCTACAACCATATGGGACAGGGCATTCATCATGCCAC
TTCTTCATTGTTAACATCCATGATGGCAGAGACATTTGAAGAACTTGCTGTTGATTATATGAAG
AAGCATTTTCCAGGACTCACAGTGAATGTAGACCACGCAGGAAGTTCAGATGACTATGCTAA
GTGTATAATAGTTTCTGGTTTGGTTTCCAAAGACATGTATAAAAGGTATGATGAAGTTTTTGG
AGACACATGTGTAGACTCAAAAATTTCTTAGCTGCGGTACAACGCTGCTGTCAGATGAAAGAT
AGTGCAAAAACCCTAGTTGGAGATTGTTTCTTAGAGTTTTACAGTGAATTTATGATGGGAAAT
CGAGTGACTCCAGCAGTCATTAAATTTATATTTACAGGATTAATAAACAGTTCTGTCACTTCTC
CCCAAAGCCTGGTTCAGGCTTGCCATGTTTCTAGCCAACAGGGCATGTACAACAGTGTCCCCT
TGGTAACAAATGCTGCTTTTACAATTCTTAGACAACAAATCTTTTACAACCATGTTGAGGATTT
TATTAGAAGATATGGACTAATAACGCTTGGGGCAGTCTCTCCATTTGGCAGACTTTTCTTACCT
AGGTTTTCAGGACTGGTCAGTTCATCAGTGGCCCTAGAAGACAGTGAAACTATCTCAAAAGC
AGCTGCTGAGATCAACTCCAACGACATTTTCTTCAATACGAGTAGTCTAAGCAACCTTGACAA
ATTAGAACAAAGTCCTGATAGTTCAGGTTTAGATGATGATAGTGTAGTAAGTACAACTACAGT
AGAGTCATCAGACTCAAAAGGGTCATCATCAAGTTTCACTTTTGACTTAAACCGTCCGCTGTC
GGAGACTGAGGTCAAATTTTTGAAGCTTTTGAGAGAATTGACATCAATAACTGCATGTGAAAT
GCTACAAGAAAAGATAAACATTCTCTACAATGATTCTAGAGAAGGCCCTTTGGATAGGCATA
ACATCTTACAAAACTGTAGATTGTCTGAGTCATGTGATTGGTTGCTTGACGGTAAGAAGAGGG
GTCTGCTTGAATTATCAAGAAGGATGTCTTGTCTGCTCAATGTTTTGATTGCAGGTTATTATAG
GTCATTTGGAAGTGAAGGAACAGAAAAACAGGTAAAGGCCTCCTGGTCTAGAGACGACAAT
AGAGTTATTGAAGATCCTATGATTCAACTTATACCAGAGAAGCTAAGAAGAGAGCTAGAAAG
ACTTGGTTTATCAAGGATGGAAGTGGATGAGTTAATGCCAGCTGTAGGCCCTGATGAGAGCCT
TTCTCAACTAGTAGCAAAGAAACTCATCAGCTTGAATGTATCAACTGAGGAGTATTCAGCTGA
GGTGTCAAGGTTGAAGCAGACTTTAACTGCCAGGAATGTGCTACATGGGTTAGCTGGCGGAA
TTAAGGAGTTATCATTACCTATATACACCATATTCATGAAATCTTACTTCTTCAAAGACAATGT
GTTCCTGGACTTAGAAGACAGATGGAGCTCTAGGCACAGTACTAATTACAGAGATAGCACAG
GTAAAATGTTGACAGGGAAAGTAGTAACCAAGTTTACCCACTGGTTGGATACTTTCTTGTCCT
GTGTTGTGAGTGCCAACCGTTCACAAGAGATTAAAGAATGCTCTCTTTTCAACCCAAATCTTA
GATGTGTGAATATAATGGTGAAAGAAAACGGTATAAAGGAACTGTCTTACATTAGGAGCCAT
TTATCAGTGCTTTCTGTTGAATTTGAGAATTTGAACCTGCAATTTTCTGATGTAAACAGGCAAA
AGCTTAAAATTGTTGAAAGTAGACCACCGGAGTGTGAATTGGAAGCAAATAAGGCAGTTATT

ATCAAATCCAAATTATTTAGTGCAGTGGAACATGTTAGACTTTCAAATAACCCTGCTGTTGTA
ATGGGTTACCTCTTGGAAGAGTCTTCAATATCAGAGGTCAAACCTACTAAGGTTGACTTTTCA
AATCTGCTGAAAGATAGGTTTAAACTTATGCAGTTCTTCCCTTCTGTCTTTACTTTACTGAGGTC
TTTACAATCTGAATCAAAAGAATTAGAAAAACTAGGTGAGCCAGTTGACATGCATCAGGTTTC
AAAATATGCTAATCATCTGACTCTGCTCTGTAGAATGATACAGCAGTCAAAACCCTCATTGAC
TGTCTTTTATATGCTGAAGGGTAGCCAAATGAATACGGAACCAACAGTTTCTGAATTAGTAAG
CTATGGAATTAAAGAAGGTCGGTTCTTGAAGCTACCTGAGATAGGGCTGGATGCTAGTACATA
TTCTGTAAGGTATTGGAAGATATTACACTGCATTTCAGCAATTGGAGAGCTCCCTTTGAGCAG
CAAAGATAAAACTTCTCTACTTATAAGTTTTTAAATTGGAAAGTAACTTCTGATTGTGTTGAT
GATTGTTGCCCATTAGAAAAATATGATAAGGCCATAGTAAGTGAATTTTCAGGACAAGTATTA
ATCCACACTTTAGCTAGTGAACTGAGCTCTGTTAGAAAGGACCAAGAAAGAGAGGGCCTAAC
TGACCTGATTGATTACATTAATTCACCAAGTGAACTGTTGAAAAAGAAGCCTTATTTAGGTAC
AACCTGCAGGTTCCAATGCTGGGGAGAAGGAGCCAAATCTGGAAAATTCACTTACAGCAGTA
GAACTGGAGAAGCCATCGGCATCTTTGTGGCTGGCAAATTGCACATCCATCTTACTTCTGACT
CACCTGGTTTGCTTTGTGAAGTCGAGAGGCAGGTCTTAAGCTGGCTTGGTAAGAGAAGAACTG
ATGTGCTAACAAAGGAACAACATCAGTTCTTCTTAGACTTCCTTCCAAACCTTAGTGAGGTGG
TGCAAAAAAACAGAGATGGTGCTATTCTGGGTGTTACAATAGACAGCACAAATGTCAGAATG
CTGAAGTATGTCCCTCCTAAGAGAAATACTCCTGTGATTAAGATCAAGAAACAAATACTAAC
AGTTAAGAAACAAACAACTTTGGATGTGGAAAGTGAGCCAAGAATAGTCTGGGGACATGGTC
AATTATCGATAGTGTATGATGAATGCGAAACTGAAACCACATATCATGAAAACTTAATCAAA
GTTAAAAAACTTGTTGATCTGGCATCAGGAACAACAGACAAACTCCCAACAGCTATCTTTTCA
GACACAAGGATCACATTAGCCAGAGTGAAGTTTAAGACAGAGCTCCTGTTGAATTCACTATGT
TTGCTTCACTGCTTCCTAAAGCACACTAGCCAAGATGCCATCCAAGAAGTTGAGAGCAAGTGT
AATGTGCTGGAAAGGTATCTAAGATCCGGTGGTGTGCAATTTAGACCGATGAGTGAGTCATTG
GACAAAAAAGTAACTAAACTTCCTTTACAGTGTCAGTCTGATAAAGACGTTGATAAAGAAAT
TAACTTCTGTGAGGACTTGACTAGAGTCTTTTCTAATGAAAATGTGCCATTGAGTTCATGGTCA
GAGGTCCAGTCTTACATAGAAGAGGTTGGCTTTGGTAATGTGCTTGTACATGTTGAGAAAAAT
CCAACAAGAAGTGATTTAATATGGCGGTTTTCAATAGACTCAATCGGCGGAAATTTCGGACCA
ATAAAGGACATTAGAACATTAGTGACTTATATGAGCACTGAAACAGTACCAAAGTTTCTACT
ACCCTTTCTGCTCTTTGAAGAGCAGCTTAAGCACCTTATAGCTGGCTGTGTAGAATTAAGAGA
TGCACTAAACAGCTCAGGGATAAATGACCGAGAAATTGCAATTGTTGCGCTATTTACTTGTTT
TTATTACCAATCTGACTCAGTTAAGAGGCAAGGGCCTGTATGCTCAATATCCTCATTTTGTAGT
TTAATAGGAGATGATTTACTGCCACTTGACAACAGACTTCAAGCCAGAGTTCTTCCTGAACAG
GACAATGTTAAACTTCACTTCAAACTCAATCTCACAACAGACAGTACATTAGGCAAAAAAGA
CAAAGCAATTCAAGCTAAAAAAATCATCAGCAGATATTTAAGGCTCATATTCACAGAAGATG
ACATGGATTTAAAAAGACTCAAAAGTGATGCCACCAAAGTAAAACTGTCGTCTGAAAAGGA
GTGTGAATTCTTAGAATTTTACCTACACAGTGACCTTTCTTATGCCTTGAACTACAGGGTACTA
TTAGAACATCTTATAGACTTAGAAGACAGAGCTAAAAAAACTGCTTGTGTGCTCATTGAAGA
ATTTATTCTGATGCTAACAGGAAGGTTGATGATCTCAAGTACAATTGACTCAGATAGCAAAAA
GACATTAGAGGATGATGCCCTCTGTTTGGAGGATCTGCTTGATAGTGACAATGAGGCATCCAG
CTCAAAGTCTGACGATGAAGAACAGATAGCACTACAAACTGGTAAATTTAACTTCAACTGGG
ATTCAGACTGAGCTCGGTTGCCATTAACAGTGACCTGCTAACTAAATCTGTCAACTTTTCTCAT
CTTTAACTAGCCATTTATTAATAGGGGTGTGGGGGAACGATATCTGCAGA

>Nairobi sheep disease virus segment S (animal origin)

ACTCAAAGACAAACGTGCCGCTTTCGCCCGAATTGCTCTTTGAACTTTGACCATGCAGAACCA
GATTGTCGCAGACAACAAGGATGCCATCCTTGCATGGCACAAAACCTACAGCGAGAAGCAC
AAACTTAAGTCTGTTTTGACTAATTCAGCTTCTTTTCTGTGAAACCATACCTGATTTGTCAGGAT
ATGAGGTTAGCATGAGGCTTGTTTCATCTGAAAGTGAGAAAGATTCAGTATATGCTTCTGCCTT
GGTTGCTGCAACTAAGTTCTGTGCTCCTATCTTAGAGTGCGCCTGGACCAGCTGCACAGGGAT
GATCCAACGTGGCCTCGACTGGTTTGATAACAACGGAGAGATGGTGAAGATCTGGGATGCAG
ATTATGGGAAACTGAGAACGGAGGTACCCAGTCCTGAACAGTTACTAGGTTACCAGAGGGCT
GCTCTAAAGTGGAGAAAGGACACCAAATATGGGATCAACAAAAACACAGCGGCATTGGCTG
CTGCAATTGCAACCGAGTACAGAGTCCCTGGTTCAATTGTAGTAAACGTCAAGGACATGCTAT
CCGACATGATTAGGAGAAGGAACAAAATCCTAAACAGAGACGGCAGTGAAGACGTTCCAAA
GAGGGGACCTGTCAGCAAAGAGCACATTGACTGGGCCAGAGATCTTGCACAGGGCAAGTTCC
TTGTTGTTTTCAACCCACCATGGGGTGATATCAACAAGGCAGGGAAGTCAGGAATTGCTCTAG
CAGCAACCGGCATGGCAAAATTGATAGAGCTGGACGGTCCCAAAGTTGCAGAGGACTTGAA
AGAGTCTCTGAAAAGCCTCGTGGCGTGGATCAATGCCCACAAGGATGAAGTGGAGAATGGCA
AAGAGGTTGTTGACGGTTTGACCAAGCACCTGCAGAAAGCCCTTGAACTAGCCAAGCAGTCA
AGTGCCATGAGAGCTCAAGGAGCCCAGATTGACACTGTTTTTAGCAGCTACTACTGGCTTTGG
AAGGCAGGTGTGACTGCAGAGATGTTCCCGACAGTCTCACAGTTTCTCTTTGAGCTAGGCAAG
GTGCCCAGAGGGAACAAAAAAATGAAGAAAGCACTGTCAAGCATGCCTCTGAAATGGGGAA
AGAAATTACTTGCACTCTTTGCTGATGATAGCTTCACTGCCAATCGGATTTACATGCACCCTGG
AGTCTTGACAGCGGGAAGAATGTCGGAGCTTGGCGTTTGCTTTGGGGCAATTCCAGTGGCCAA
TCCTGATGATGCAGCTGAGGGTTCAGGTCATATCAAGAACATACTGAACTACAAGACTGACA
CCCAGGCTGGTAACCCCTGTGCCCAGAACATCGTTGCCTTGTTCAACATTCAAAAAGCAGGCT
TCGACATTGAGAGCATGGACATAGTTGCCTCAGAGCATCTGCTGCATCAGTCACTTGTTGGAA
AGAGGTCCCCTTTTCAGAATGCCTACAACATCCGAGGGAATGCCACCAGTATCCAAATCATCT
AGCCGAACCGCTACCTGCTGCTTGCTTCTTTCTGTTTCTTTTATTTCTGCTTTTATTAACAAGGG
GGCTGTGCGGCAACGATATCNTGCAGA

>Nairobi sheep disease virus strain segment L (animal origin)

TCTGCAGATATCAGTACCCCCAGGTTACAGAAGCATGGGTTCTGAACAGCATTCCTTGGGAAG
AGGTTGTTCCAGGGCAGTTCACTGCAAACCGGATTTTCAGGTCACGGATTACTTTGAGATAGT
CAGACAGCCTGCTGATAGCTGTTTTACCACAGCATCGCTGCACTTCTTTAAGTTGCTAAATTAA
AAAGGANNTTACATACAGACCTGTGAAGCAGCACCTTGAGTTGGCAGCGAGAAGATTCTTTG
AGGAGGAATCCGAAGCCAAAGGACTAGGCCTGAGTCTTGAGAAGTATTTAGAGGTTGCTATG
TGTGACAATGAGTGGGGAGGGAGTCTGGAAGCTTCGATGTTGGCAAAGCACCTAGACATCAC
TATTGTCATATGGGTGATAGAAGGTCCTAGCAGAGTGGCTGCAGCAGTGAAATTTGGACCTGG
AGATGTTGCTGGCGCAATAAATCTCCTACACACCGGTTACAATCACTTCGATGCCTTAAGACT
GTTAGTTGATGACAGCCAAGTCAGCCGTCAACCTAGAGACATCACAGAACGCATAGAAATAG
TTGAAGAGGTGCTGTCTGAAGACAGAGAAGAAACCTTCTTTGAAGAAGATCTGCTCAACTTTG
CCACCACAGAGACTGTAGAAGAGAAGCTGACCAGGCAAGATAAGAAGGTTCAGGATGAAAT
GCAGCGTAGGGCAGCATTACTAGGGAAAATCATAAAGAAAGGTGAAAACATCCCTGTGAGA
GTAGGAAGGGTACTTGACTGTCTGTTCAACTGCAAATTGTTTGTAGAGCTAAGAGATGGACTA
CTGGTGTTAAAGCCAGAGTCGAAAGAGGATCCAAGCATGGGGTGCAGTCTGAGGCAGCTAGG
CCACAAACTGCTAACAAGAGACAAGCAAATAAAACAGGAATATGCTAAGTCTAAGCTATAC
CTGACAAAAGATCTTTTGGACCACCTAGATGCTGGTGGACTCTTGCGCTCAGCATTTCCAGGG
ATGGGATTAGAGAGAAATCTGCAGTTCTTGCACTCCGAGGTTCTACTTGATGTGTGCACAGTG

GTTGTTGCTGTTCTACTGTCCTCCTTCCTGTATGGGTCAAACAACAGAAACAAGAAGACATTC
ATAACAAACTGCTTGCTGAACACGAGCCTTTCAGGGAAGAGAGTCTTCAAGGCATTAGGAAA
GCTGACAGGTACCACTCTTTACAGAAGTCCTAGGAATGCCTTGTCTCATGTGTGTCAGACTCTT
TATGGCAAGATGATGGGTAGGCTACAGAGCTACATTTCAGTCATGAGTCCAATAAGTTTGTTA
GCTCTACGAAATCTCGACTTTGACAACATGTCTGTTAAAGACTACATGGAAATGCTAAAAGA
GATGTCAGTGATAGACAATACAGATGTTGACTACACCCACAGAGAGATTGCTGACCTCAATC
AGCTCACAGATAAGCTTCAAAAGTTATTTAAAGAGGGAAGGGCAGATGAGCTGAAGAACTG
GTACAAAGAGGAAGAATTGACAAAGCGGTCATTGAGAAGTGTGCAAAATGCATCTGAGTTTT
TAATAAGTGACTATTTTAAAAAGAAGGACATTATGAAGTTTATATCAACAACAGGCAAGGGC
ATCAAGCACTGCAAATATTGGCAATGTCCTATCTTACGCACATAATCTATACTTGAGCAAAGA
GAGCTCTAAAGATGACTACAGAAGACACAACACAGCTTCTGATTGAGATAAAGAGATTATAT
AGGCTTCAAGGAGAACAAAGCATTGAGCCAATTGCAACAATATGTGATAAGCTTGAGGAGCA
ATTTAGAAAACTATTCAAAGAGTTGCCTGAAGAATGTTCACTNGAGTGTCAGACTCTGTTCAA
TGACATTAGAAACTCTGGTAGTCACAGTACAGCATGGAAGCATGCACTGAGGCTTAAAGGTA
CTGCTTATGAAGGAATGTTCTCTAGGCAATATGGATGGTCATATATTCCTGAAGATATAAAGC
CGAGCTTGACAATGCTGCTTATACAGACTCTGTTTCCTCATAAGCTTGAGGAATACGTTTCTGG
ATCGAACTCAGCTCCATCCAGAATTTAGGGACCTGACACCTGACTTCGCCTTAACTCAGAAGG
TGTACTTCAAAAAGAACAAGATCGTGGGAATTCAAAACACCCAGTTAGTAATTGACTCAAGC
TTAGAGGGGTCTGTTGAGGCTGTTCCCGTGGTGGAGAAGAAGATGTTCCCTCTACCTGAGACT
CCAGTTGACGAAGTACACTCCATCCAGAGGATTATGAAAAGTTTCAGAGACAAAGTTGAAAA
TGACAAAAAGAGAAAGGAGGAAGAAGGTGACAAAGCACCAGCAAGTGAAAGCATGGAAGA
CCAGAAAGGTGGTAGTGTTCAAGACCAAGACAAGCCTGCTCCCATAACCAAGAAGGCAATG
CAGGAGGAGTCAGAAAGAGCAGGAGAATCCAAGTCCAACCTGGAATCAAGGGGAGCTGACA
AATTACAGCCAGTTTTAGCATCAACAGGCCCCCGTGGCATGGCTCAAAGTGCAACCAGAGGA
CCACTAACATACAGTGACAGAATTCTTATTGATGAAAACAGTACAGAACTAACAGAAGAAGA
AGAGTTGGAAAAGAGACAGATACTACTGGTTGAAGTTGGTTACCAGACCGATGTGGATGGCA
AAATAACAACAGACTTCAAGAAGTGGAAAGACATCTTGAGGCTTTTGGAAATGTTAGAAATT
AAGTGTTCTTTCATAGCCTGCGCAGACTGCACATCAACACCTGCAGACAACTGGTGGATCTCA
GAAGACAAGGTACGTTGTCTAAAGTCCTCCATAAGTCACCTGTTTAGCAGCCTCACAAAGAA
CTCTCCTGCGGATGTCACGGACATAGTGGTTGGCTCTATCAGCACACAGAAGGTGAGAAGTTA
CTTGAAGTCTGGATCAGCAACCAAGACACCTGTCTCCAGCAAGGATGTTAAAGAAACTTGGC
AACGTATGCGACAGAACATAATAGAAAGGCCTACAGGGGCCATCATCAATTCAGGCCTTGAG
AGTGCCATGAGACAAGGATTAGTTGATGGAGTTGTGATGTCAAAAGAAGGGTGTGTGAAAAC
CATTGAAACTTTAAAACAAAACTGTGACAGGATCACTGATGAGTTTGAAAGAACAAAGTACA
AACATGAACTAAACGAAAACAAGACAACCAGTGAAAAGCTACTTCTGGGATGGTTGTCCGA
GGATCTCCAGGGTTGCAGATGTAGCAACTGTTTAAACATCATAAAACAAACAGTTGAGAGTA
TAACAGAGAACTCAGATAGGCTTGAGTATTTAGCAAGTAGCACTATCCTCAAGTCGCACTGCC
CTGATTGCCATCCTAGAGGGGTTAGTGTTAGCAATAGCACCAATATAATGAACAGACTGCCTG
GGCTAGAAAAGACCCAACATTCCGACAACAAAGGCTTTGAAGACACAAATGAAGCTCTGAC
AGATCTAGATAGAGTGGTCCGGTTGACTTTACCCGGCAAAACAGAAAAAGAGAGAAGAATC
AAAAGAAATGTCGAAACACTAATCAGGCTAATGATGCAGGCGTCAGGCCTAGAGTGCATAA
AACTCCCTTCAGGGCAGATTATCACCCACAGACTGACCAGGAAGATCAAGCAAGAAGATGA
AAATGTTTTATCAGACAAACTGACAGAAAGGCTGGAAAGAATTAAGAAGGAACTCTCTGATG
CAAAATTGAGCAGTTACTCAGAGTATGTGAAGAAAACCTTAGGGCACTCCATACAAAGAGTG
GATAAACAGAAGGAATCCAAGTGTTCAGTGCCTAGAGCATGGCTAGAGAAGCTTTTAAGAGA
TTTGAAAGTTCCTACCAAAGATGAAGATATACTAACAAATATTAGAGAGTCAATGTCAAAAA

AGACTAACTTCATTAAAAACAATGACAGGCTTGTGATTAGATCTGAAGAAGAGTTAACGAGA
TTTGTTGAAAACAGGTCTGTACAGTTAATGCCTGACAAGTCTAAGAAGCTCTTTCAATCTGATT
GTATCCTCTTCAAAGAAGTCACTGCTGAAGCAATGAAGCGGTATTATTCAACACCGTATCGCA
GGAGTTCCGGCAGATGATTGTGCTACTAATAAACTTCTTATGCAGATTCCCTTGGTTCCAAGA
GGTTGTTCTCTACGGGAAAATCTGTGAGACTTTTTTGAGATGCTGTACTGAGTTCAGTCGTTCT
GGAATTAAGCTTGTCAAGGTGAGACACTGTGATTTAAACCTAGCAATAAAGTTGCCCTCTAAC
AAGAAAGAAAATATGTTATGTACTCTGTATAGCAAGGAGATGGAACTGCTGAAAGGACCCTT
CTTCCTCAATAGAAGACAGGCTGTCTTAGGTGCTTCGTATCCATACATAATCATCACCCTCTAC
ATGCAAGTTTTACAACAGCACAGGTGTCTTGAGGTCTTAAGCACAGTTGGTGAAAGAACATTT
GAAAACATCAAGACTTGCACCTCAGACCTGATAGCTAGCCTATCATTAGAACTGACATATGCT
GTCAATGGGCAGTTTGAGAAGGCTTATGAAGTTAGAACAAGGCAGTGCAAGCTAGGAGGTAA
CTTTTTAAACAGAAGCAGCAGAGACCACTTCATTACTGTGATCTCAGGTTTAAACACTGTTTAT
GGACTTATTGTAAGGGATAACCTTCTTGCAAATTCCCAACAACAAAACAAACAGCTTCAGAT
GCTAAGATTTGGCATGTTGTCTGGCTTGAGTAGGCTTTCGTGTCCAAAGGAACTAGGAAAAAA
GTTTTCCACGAGCTGCAGAAGACTCGAGGACAATATCATGCGGCTCTATCTACAAAGCACTGT
TTACTGTTCAAACAGGGACGTTGAACATAACATCAATGAGTGGAAGTCGAAAGATCTATGTC
CTGAAGTTACAATCCCATGCTTCTCTGTTTACGGTGTGTTTGTGAACAGCGATCGTCAGTTAAT
CTTTGACATTTACAACGTGCATATCTACAATAAAGAGATGGACAATTTTGATGAAGGATGTAT
AAGTGTGTTGGAAGAGACTGCCGAAAGACACATGCTCTGGGAAATGGATCTACTTAGGTCAT
TAAGCAGAGAGACAAAAGATGAGAGGTCAGCAAGGCTTCTATTAGGATGCCCAAATGTCAG
GAAAGCAATCGACAAGGACGGCAACAAACTAAGCAAGGCAGGTAGCACATCTCCCGATGAC
GGCGACAGTGACTCATCAAGCTTAAGTGGGAGACGTTCTTACTGCTCTAGTAGAGGGAGGATT
CAGAGCATATTTGGCAGGTACAATTCCAACAAGAAACCTTTTGAGTTTAGACCAGGTCTAGAA
GTTAGAGCAGATCCAATGAATGATTTTGAGCAAGCAGTCACAGACACATCGCAATATGCAGA
GTACACACCGAATCAAGAAAGCTTAATGAAAGATTTTATCCAAATTATAAGGACTAATCCTA
GTCACACAATGGGCTCTTTTGAGCTCATTCAAGCAGTAACAGAATTCGGCCGAAGCAAGTAC
CCAGCAGAGAACATAGAAAAAGCTAAAAGGGACCCAAAGAACTGGGTGAGTATCTCAGAAG
TAACAGAAACAACAAGCATTGTTTCACAACCCAGAACACACATAATGCTCAAAGACTGCTTT
AAAATTCTTCTCGGTACTGAGAATAAGAAGATCGTCAAGATGCTAAGGGGAAAGCTGAAGAA
GTTAGGTGCTATAAACACTGATATAGAAATTGGAAGGAAGGACTGTCTGGACATGCTAAATA
CCGTTGAAGGGATATCAGAAGAGCAAAGGAAGAACATTGTTAATGGAATTTTTGAGCCTTCT
AAATTGTCATTCTACCACTGGAAGGAACTAGTCCAAAAAGATGTTGAGGAGGTGCTGCTGAC
TGATGATGGCAACTACATCTTTTGCTGGTTGAAAACACTGTCATCAATGCTGAAAGGGGCTTT
GAAGAAAGAGCTCCGATTCATGAACAATGGAGGTGTGCTAGAACTCAACAGTGGATTCTTCA
GTGAGGATGAATTTGAGGAGCTGCTAAACGTCAAAAAAGAACTGACTGGAAACATGAGTCTA
GACAGAGAGTTAAACACAGAGTTACTTTTGGCTTCATGGCTGAAGTGTGTCTACAAGCCCAAA
GAAGGGGCCTCAATAGTGCAAGAAGGTTTAGAAGCACTGAAGGCGATGGCAGGAGAACTAT
ATGAAATCAGATTGCAGCATTTGGAGCTAACCAGAATGAAGAAGGACAATCCTAGTGTAAGC
TTCACCAAGGAAGAAGTTCTTGTGAAAAGGCTTGAGAAGTCATTCCTGAAGAAATTTAACAA
GGAAGCAATGAAGTTTGTTAACCTGGTGTTCTTCTGTTCACTCTCTGCCCCGTGGTGTGTTCATT
ACAAGTCTTTAGAGTCCTACTTGGTCCGTCACCCAGAGATCCTTGAGCTAAAGGCCAAAGGTG
ATTTGGGATCTGTGATTTTAGACCTTTCGGTTGCATCGGCTATATCAAGGCTAGTCCAACAAGA
AACAAACAGTGAGCTAGATTCAGAGGTGTTAAATGAGATGAAAGTGCGCTTTGCTGTCAAGT
ACTTTGTAACACTGTTTACAGCTAATGGAGAGCCTTTCTCACTTAGCCTGAATGATGGAGGTCT
AGATGAGAATCTACAGAAAACCACAGACGAGAAGCTGTTGCATCAGACAAAAGTAGTATTT
ACTAAAATCGGATTGGCAGGAAACAATTATGACTTCATGTGGACTACGCAAATGATAGCAAA

CAGCAACTTCAATGTTTGCAAGCGTCTGACTGGTAGAACAACTGGTGAAAGGCTTCCCAGAA
GTGTGAGAAGCAAGGTAATTTATGAAATGGTGAAGCTTGTTGGAGAAACTGGAATGGCAATA
CTACAACAGCTGGCCTTTGCTCAAGCCCTTAATTATGACCATAGATTATCTGCAGTANTAGCA
CCTCAGGCGCTGCTTGGAGGGTCCAGGGACCTGCTAGTACAAGAGACTGGAACGAAGGTCAT
CCACGCCACAACAGAAATGTTCAGTCGAAATTTACTGAAAACAACACAAGACGATGGTTTGA
CAAACCCTCACTTGAAGGAAACCATACTGAATGTTGGACTGGATGCACTGAGCACGATGCGC
TTGCTAGACGGAAGGCCAGTGTCTGAAGACAGCAAGCTTCTGAACTTTTACAAAGTAATCTGT
ATATCAGGAGACAACACAAAGTGGGGTCCTATACACTGCTGCTCTTTCTTTTCTGGTATGATTC
AGCAGCTTTTGAAGGATGTGCCAGACTGGAGTTCATTCTACAAGCTTGCTTTCATCAAGAATC
TTTGCAGGCAGGTTGAGATTCCTGCTGCCAGTGTAAAGAAAATCCTGAACACACTTCGGTTTA
GACTTAGTAACAAAGGAGGTGTTGAGTCAAAGTCAGAAGAGGAGCTCAGGAAAGAGCTAAG
CGATAGCACTGAAGAATGGGGAGACAATGACACTGTCAAGTTTCTGATCACAACCTATCTAA
GCAAAGGAATCATGGCACTCAACAGCTACAATCACATGGGTCAAGGGATACACCATGCCACC
TCATCATTGCTGACTTCGTTAATGGCAGAACTATTCGAAGAACTAGTGATAGACCATTTTAAA
AGGCATCTACCACAGCTCACTGTCAACGTGACACATGCTGGAAGCTCTGATGATTATGCGAA
GTGCATTGTGGTCACTGGGCTTCTGCCTAAAGAACTCTACGACAGGTATACTGAAGCTTTCTG
GAAGCATGCATGCAAAATAAAGAACTATACTGCAGCCGTCCAAAGGTGCTGTCAGATGAAGG
ATAGTGCGAAGACATTGGTAGGAGATTGCTTCCTCGAGTTCTACAGTGAGTTCATGATGGGCA
ATAGAGTAACTCCAGCTGTGATCAAGTTCATATTCACTGGCCTCATAAACAGTTCAGTGACTT
CACCACAGAGCTTAGTGCAAGCCTGTCATGTATCTTCTCAACAAGCAATGTATAACAGTGTCC
CGTTGATGACTAATGCAACCTTCACTCTTCTTAGACAGCAAGTTTTCTTCTCACATGTGGAGGA
TTTTGTGCGCAGATATGGGCTGATTACACTAGGCACTGTGTCATCCTTCGGTCGCCTGTTTGTTC
CCACGTTTTCTGGCCTAGTTAGTTCTGCGGTTGCCTTAGAAGACAGCGAAGTCCTGGCGAAGT
CAGCTGCTGAAATCAACGAAAACAACATTTTTCTAGATAGCAGCAGTCTCTCCAATCTAGACA
ACCTAAAAGACACCGACAGTGCTAAAGCTAGGGATGATGAGAGTTCCATAAGTGAGACCAC
AATTGAATCCACTGAATCAGGAAGTTCCTCTTCTAGTTTCACTTTTGAGCTGACAAGACCGCTA
TCTGAAACAGAACTGCAGTTTCTCAAAACATTAAAGTCGACCACTTCTGTAACTGCATGTGAA
ATAATACAGGACAAGCTGACTCCACTATATGCAGACAGTCAAGAAGGTGCGTTAGATAAGTA
CAATGTGCTGTACAACAGCAGATTGACTGCTTCTTGTGATTGGCTCAGACAAGGAAGGAAAA
GGGGACCTCTTGAATTAGCTAGGAGGTTGCAATGTATCTTGAACATTCTGATTGTGGGTTATTA
CAGATCATTTGGTAGTGATGGGACAGACAAGCAAGTCAAGGCATCCCTCAATAGAGATGACA
ACAGAGTCATCGAAGACCCCATGATTCAACTAGTACCTGAGAAGCTAAGAAGAGAGTTAGA
GAGATTAGGAGTCTCCAGAATGGAAATCGATGAACTTATGCCTTCCATCAAGCCTGATGAAA
CTCTCTGTCAGCTGGTGGCCAAAAAACTCATCAGCCTGAACGTTGCAACAGAAGAGTACACT
GCAGAAGTCTCTAGGCTGAAGCAGACCTTGACAGCCAGAAATGTCTTGCACGGCCTTGCTGG
AGGCATCAAGGAGCTGTCACTTCCAATTTACACCATCTTCCTCAAGTCATACTTTTTCAAGGAC
AATGTCTTCTTAGACCTAGAGGATAGATGGTCAACGAAGCATAGCTCTAATTACAGGGACAG
TTCTGGTAGAATGCTAACTGGTAGAGTAATCACAAAATTCACTCATTGGTTAGACACATTTCT
GAACTGTACGGTGAGCATTAACAGGACACAAGAAATCAAGGACAATTCTCTTTTCAACCCTG
ACCTCAGATGTATAAACATTCTAGTGAGAGAGGACAATGTGAAGGAGATGTCAATTGTTCAA
AGCCACCTTAGGGTTGTCACTTCAGAATTCAACAACCTCAATCTTCAGTTCTCTGACTGCAAC
AGACAAAAACTGAAGGTGGTTGAGTCTAGACCGCCTGAATGTGAGCTAGAGGCCAATAAGG
CGGTAATTGTAAAGTCGAAGTTGTTCAGTGCTGTTGAGCAGGTCAGACTTGCAAATAACCCTG
CAGTGGTTATGGGCTACCTCCTTGAGGAGTCATCAATCTCAGAAGTGAAACCCACCAAGGTA
GACTTTTCTAACCTGTTGAAAGATAGATTCAAACTGATGCAGTTCTTCCCCTCCGTCTTTGCTTT
GCTGAAGCACCTTCAGTCTGAGTCGTCAGAGATGGAGAAACTAGGGGCACCAGTGGATATGC

AGCAGGTGTCAAAGTATTCAAACCATTTGACACTATTGTGTAGAATGATACAGCAGGCTAGG
CCTTCGCTGACAGTTTTCTACATGCTGAAAGGCAATCAGATGAACACTGAGCCTACAGTGTCT
GAGCTGGTCAGCTATGGGATCAAGGAGGGCAGATACCTAAGACTGCCTGAAATAGGTCTTGA
TGCCAGCACATACTCTGTTAAGTACTGGAAAATACTTCACTGCATTTCTGCCATAGGTGAATT
ACCACTTAGTGACAGAGATAAAACATCCCTACTTATAAGTTTCCTCAACTGGAAAGTTTCATC
TGACTCAATGGCACAAGATTGTCCGCTTTACAAACAAGAGCATGCTGTTATCAGTGAATTTGC
AGGACAGGTTGTTGTGAACACTCTTGCTAGTGAGCTCAGCTCTGTTAGGAGAGATGCTGAGAG
AGATAGCCTTACTGACCTGATTGATTATGTGAATTCACCTACAGAGCTACTCAAGAAAAAACC
CTACCTCGGTACAACATGTAAATTCACTACCTGGGGTGAAAACAACAGGAACGGCAAGTTTA
CATACAGCAGTCGGTCGGGAGAGGCAATTGGCATCTTCATTGCAGGAAAACTCCACATACAT
CTGAGCAGGGAATCAACTGGTCTGCTATGTGAGGTGGAAAGAAATGTGCTTGGCTGGCTTGG
GAGACGTAGGACAGATGTACTAACAAAGGAGCAGCACCAACAATTTTTAGAGTTCCTTCCTA
CGCTAAGCGAGGTGTCACAAAAAAATAGGGACGGCACCACACAAGGTCTATGTCAGGACAA
CACAAATGTCCGTATGCTGAGATTTGTGCACCCAAAAAAGAACACTCCTGTTGTCAAGATCAA
GGGGCAGATTCTGACAGTGAAAAAACAGGTTAGTTTTGAGGCAGAGAGTGAGCCTAGACTCA
TGTGGGGGCATGGTTGTGTCTCAGTAGTTTACGATGAGTGTGAGACTCAGACTACGTACCATG
AAAATTTACTCAAGATAAAGCAGATGGTTGATAGCACAACTGATAGAGCTAGAAGCCTCCCA
CAGTCAGTATTCTCAGACACCAAAGTGATTCTTGCCAGAATCAAGTTCAAATCCGACCTTCTG
CTGAACTCACTTTGCCTGTTGCACAGCTTCCTAAGACACACAACTACTGATGCTGTGCTGGAG
GCAGAAAGCAAGTGTGCACTGCTAGAAAGGTACCTGCAGTCAGGAGGGGTGAGAGTAAAGT
CAGCAGATGAAACTTTAGAGAGAAAGTTATGCAGCAAGGTGATAGAATGTAAGCTTGAGCAA
ACTCTTGATGAAGAAATTGAAGTCTGTGATAGCCTCAACAAAGTGTTCTCCGAGACTCCTGTT
CCCGTCAGTAGCTGGTCGGAAGTGCAGTGCTACATTGAGGATGTGGGCTTCAGCAACATCCTA
ATAACACTTGACAAAACAAGCACTAAGGGAGAACTGATCTGGAAATTTTCTCTAGACAATAC
AAGCAACATAGCTGGTAGCATCAAGGACATAAGGTCGCTTGTTTCCTACATAAGCACCGAAA
CTATACCAAAATTCTTGCTACCTTTCCTGCTCTTTGAGAACCTGCTCTCCAGCATTCTCAAGCA
AAGCTTGACAGTGAAGGAGACATTACATGCTACAGGCATCTCTGACAAAGAAATTGAAGCAG
TTGCCACCTTATTTGCTTTCTGCTTCCAAAACGACAAGGTCAAAAGAAAAGGCCCCCGATGTT
CAATGTCCTCTATCCTAAACTTAACCAAAGGTGATTGGGTGGAAGTAGGGCAGAGAATGAAA
CTTCAGGCACATCTGGATTCGGACACTGTCCGGCTGAGTGTTCAGTTAGCAATCTCTACCGAT
GCAGATCAAACAGCTGATAAAAAAGCTCGAGTGTCAATGGCAAAGAAAGTGATAGCTTCTCA
ACTAACACTACTACTGCAAGAGGATGGTATTGACATTAAAAAACTTAAGGACATAGCTGTTA
ATGTTCAGGTTAGGAAAGAAAAAACTGGTGAGGTACTAGATTTTACACTTCTTGATGACCAAG
CAGGTAACCTGAACTATATCGGAGTGTTGGAGACAATAATGGATAGAAGAAAGAAAGGTCCT
GCCATCAGTGCACTTGAAGACTTCTTTCTTCTACTAACAGGCATGGCCGAAAGCAACATACAA
CAGGACACAGCAATCAAAAGTGAAGAAGCTTGCAGTGACGACATATGTCTCGAAGACCTTCT
TGAACCCAGTGAGGAATCATCCAGCAGCTCCGGTGCGCAGGACAAAGAAGCTGTACCCGAC
AGAGTAACATTCAACTGGGATTCTGACAGTGATTGATCTTCAGTGTGTATACTAAAAGTTTAA
ATTAGTAGGGGTGTGGGGGGAACGATATCTGCAGA

>Nairobi sheep disease virus segment M (animal origin)

TCTCAAAGAAAGACTTGCAGCTAGCAGGTTAATCTCAAAGGCAATCAAGATGGCATTAGTGG
CAAAGGGTTTTAATCCTCATACTACTACTATATATGTTCTAGGAGCCTCTGAAGTCAACCTTGA
ACTCCCCTAGTTGGAACGGCACTGAAGGGACAGAGTATGCTGTTGAGAGGGCTTGCCCACTG
AACCTCCTTCAGGAACAGGAGCCCAAACTAGAAGCAGTTAGCACGGAAACCCTGGACAACC

AGCACCTCCCAAGAGGCTACTGAGAACACCATCGAGAGCCCTGATGGAGGGGAAACAATGG
CACCCACAACCCCAGCACCTGTGACTGATTCAGCCAGTAAACTGGAAGAGAGGAGCCAGGC
ACAACCCAGCAGGCCCAGTCAACTCAAATGCAGAACAGCCAGGCACCGATAGGAATGCCTG
AAGCCTGGTCGACAAGTCCCTCCAGAGTTGGTAGAAAACCTCGTTAAAGTGCTAGCCCTGAT
GTGTCTGAGCCAGACCAGAACACAGAAGCACAAACACATCCACCAGTCTGCCTTGACGGACG
GCCTGTAGAAGAAGAAGAACACTCAGTTGGCACAAGAGATGTAGCTAGTGATCTAAGAGAA
GTACAATCAAATGAAACAACTCTTGAGCCAAAGAATACTAGCAAAGATGGGTATGGCTGTAG
ACTTTACCGATGATGAGTTGGACACTTGGTGTTACAGAAGATACAGCAATTGTAGCTCAAATG
ACATTGAATCAAGAATTAGGGACTTCTTCCTCATTACTGATAGGTCTGAGTGCTTTGATGAAGT
CCTGGTGAAAAGATTGTGTGAGACCACCAGCCCGATTATAGATAGGGCTTGGAAGATAGCAG
GACTCAAAGAAGAAGTTGTCTTGAGGGAGATGGGAAGAAGGATCTTTAGGTTTTTACACCTG
CCCTAAAAGTGACATGCATGTCTGGCTCATTAAATCCCTCTAATCAATTTGTAAGGTTCTACAA
TCCCACTTTAGAAAGGACATCAGGCCCTACCATCCAAAACTACGATGGCATGCACTGTCTAA
ACATAGAGAATGGGCTCATCAAACCTTCCAAAGTGGTTGTTATCAATGTTCTGATGACCACAG
TTGACATCAGACTGGAATCCTGCCGAGCATTCATAAATGCTCAACAGTGTACTTATACCCAAC
ATGCTGATGGACTGGTGAGGGTGCCTACCTTTGTGGGTCCTCATGGTGAAAAGAGGATTATTG
GTGCCTATACCATGAGCTTCAACCTAACTGATGAAAAGAACAAAGCCTGCACAATCAAGACA
ACTTGTGTTGTTAAGGGAAAGGAAGTAAAAAAGGGACAAAGCCAACTTAGAGGTTTTCCTAC
AACTATCAGGCTCTTTAAGTCTGTAACAGGAAAGAGAAGGCTCATGTCAAATGAAGAAGAGT
GGACTGAATGCGGTTCAGGGACGCAACTAAACATGGCAACAGCTGTCTCAGTGCACAACGAC
AAGCTAGGAGGACCTGGGAAAAAGCTCACTATCTGCAATGGAACAACAGTCTCAGACATTGC
TCTAAACGAGGGTCTTGGGTGCTACACAATAAACAAGATCATTACAGGGAAGGTCTGCAAGA
CAGGCAATACAACAGGTTCTTGTGAAGTGCAACCAGAGCTGCAGAAATGTGAGACAGGAAA
ATGCATACTGATCAAGCAAAAGAACAAGGGTGTTGTGAAATTGAAGAGGGGCAAGACTGTG
ATTATAACGGAATGCCAAGGAAGCTGTCTTTTTGCAATACCACAGGACACGGGAGACATTAC
AATTGACTGCTCTGGTGGAAGACAGCATTACCTGGAGATCAACATTGTAGATATACACTGCCC
AGGGAAGGATAAATGGAAGGGCTTCATGCTCTACATATGCAGAGTCTCAAGCAGACCACTCG
TTGCTCTAACATTTGGAATATGGCTTACAGCAGGCTACTTGATAACTTGTCTGGCCTCCTTCAT
CCTGTACAATGCCATACTGCTTATTAGCATTGCAATCAAGAAACTGAAACAGAAGAGAGAAA
AAAAAGGTGACCTATGTATAAAGTGTGAGCAGCACTGCATGAACCTGTATGATCAGGAATTG
CACGAGCTAAACTGCAGCTTTAACCTATGTCCTTACTGTGCTAATAGGCTCTCAGACGATGGG
CTCCCAAGACACGTGCCCAGATGTCCCAAAAGGGGAGAGAGGTTAGAAGAAATTGAGCTCT
ATATCAATTATACAAGAGTACCCTGTCTTCTGCGGTGGGCGCTCTCAACTTCTGTCCAAGTTGG
CACAGTTATTAAAAGATTGTCTTGGTTCGGCGTGCTACTAACACTATTCTTCTTGACGATATCT
CCTGTGCAGGTGCTGCTTGTCCAGGTTGGTAAATCCTTTAACCTTAGCAATACGTAACTGACA
GCAACGGATNATCTGCGGAGCTCCGGCCGGTGCTGCATCAGGATCTGCAGAGAATCCCAGGT
GCTGCTGTCCAGGTTTGTGTAACCTTTTAACTCAGCAATACGTAACTGAACGAAGTATTATTCG
TTCAGTTACGTATTGCTAGGTTAAGACTACTTTCTGCCTTTGCGAGAACAGTTTACCTATTGCT
AAGGAGCTACTTCGTTCAGTTAANGTTACACAAACCTGCGGAGGTAAAAAGAGATTCTCANA
AGAAGCTGCAGATCAAGAGCATGTGCCTGAACAGGATGAGTGCCTGTGTCCCGGAGAACATA
GGGCTGGAAGGAGGCTCCTTTTTCTGAGCGGCCTTCAAGATGCTGCAAGGAGGATGACAGAG
TCTCACAGACTACTTACAAGTGTCTCGATTGATGCGCCCTGGGGTGCGATAAACATTGAATCC
ACTTTCAAGCCTCTTCTTGCAGCATCCAACATTGACTTGAGTTGGAACTCAGCAGAAGAACAG
GGAGACAAAATTGTACTGTCAGGTAGATCCACAGGTATCATAAAGCTACAAGAGAGGACTGG
TCTGATGTGGAAAATGTCATCTGAAAAGGCTTCTGAAAGTAAAAATCTTCTTGTGTCTATAAT
GGACTTCTCACAGCTTTACAACTCAATTTTCCAGTACATCACAGGTGACAGAAGCCTTTCGGA

ATGGCCAAAGGCTGTATGCACAGGAGAGTGCCCTGACAGATGTGGGTGTCAAACCTCCACTT
GTTATCATAAAGAGTGGCCCCACACCAGGAACTGGAGATGTAACCCTACGTGGTGCTGGGGC
ATCGGAACTGGGTGTACTTGTTGTGGAATGGATGTTGAAAGGTACTTCAACAAGTACTTTGGG
GTAAAGTGGGCATTGGAGTATGTAAGAACTGATGTGGTGGTTTGTGTGGAGCTGACCAATGAG
GAAAGACACTGTGATTTAGTTCAAGCTGGCAGTCGCTTTTCTATAGGGCCAGTCTCTGTGACA
CTGTCAGACCCTCAAAATGTGGTAAACAGACTATCAAGTAGCATAATGACAATACAGGAGAT
ATCAGACAACGGGATCTTAGATGTCATGCATGTTAGTAAAGTAATTTCAGCAGAAAATGCTTG
CAAACTTCAAAGTTGCACACATGGGAGCCCAGGAGATCTCCAGATCCTTCACACTGACAACT
TAATAAAAGGTGAAATGTCATCCGGCATAAATCTTGCTCACCTGGACCCTCAGGTTAACACAA
GTTGGATGTCGTGGGAGGGTTGCGATCTAGACTACTTCTGTACAGTGGGAGATTGGCCTAGCT
GCACGTATACAGGAGTGAACTCCATAAATACAGACAGCTTCACAAACCTCATAAACACAGAA
ACTGATTATACAAGCAAGTTTCATTTCCACTCAAAAAGAATTTCTGCTAGGGGTGACACTCTG
CAGATGGATTTAAAAGCAAGACCAAACGTTGGAGGTGGGGAAATGACAGTTCTGGTGGAAGT
GAATGGCCTGGAGCTGCACTCAAAAAAAATAAGCCTTAAAGGATTAAAGCTGTCTAACCTCA
AGTGCAGTGGATGCTTTGCTTGTAGTCCTGGTCTGTCGTGCACAGTGATTGCCAGATTAGAATC
ACCAGATGAGTTCACTATACATTTAAGAAGCACCAGCAGAGATGTGGTGGTTGCTGAAACCA
GTGTGACAGCTAGAAAGGCAGAAACGGGGGCCAAGAGCAGTTTTAGGGCCTTTGCTGTGAAA
GATGTTAAGGAGATATGTCTAGAGGTGGTCGAGAAGGAATATTGTCCGTCTTGCTCCATAGAA
GACTTGAAAATTTGCATTAGTGTCACTCTTGAGCCTCCAAAGGACATTCTCATTGAGCATAAA
GGCACCATTGTACAGCATTACAATAAAAGCTGTGATGAGGGCTACAACTGCTGGATTGGATCT
GTCTCTGGGTTTTTTGTAGGGGTTAAGGATTTTTTTGAAAAAAACTTTGGGAGTGTGATCATTG
GAATTGTCAGCACAGTTCTTCCCTTGATACTCACTGTTTTGTTCTTTGTCTATGGAAGAAGGCTA
TTTTGTCTGTGCAGGTTATGTCACAAGAAATGCTGCAGAGGTTCATCTAGAGGAAGAGACGGT
TACAGCAGACTGTCACAAGAAGAAGAAATAAAAGAAATAATAAAAAAATTCAACAGAAATG
GAGAGTTACTGGGAAGGGGCGAGAACGATAAAAGAACTGTAGCAAGGATGTTCATGGATAG
CCAAAGTGCAAGAAAAGCAGTTAAAGAGGTTGCCTAGGCTCAATGGCATGCCTGTTAAACTG
CTTCTCAAACTTAACCCAGCCACTTATATTCTCGATTCTATCATGCATGCTCACTATCCGATGC
CTAATTTTCCACTTAAATCTGAGCTTTAATAAGAACTAGATATTAATAACTGCTGTGCCGCCAC
GATATCTGCAGA

>Middelburg virus (animal origin)

ATTGGTGGTTACGTACACCTGCCAGCACTCCGCACTATCCGAGCGATCCAAAATGGCGCGCCC
TGTTGTGAAGATAGACGTTGAGGCCGAAAGCCCATTTGTCAAGTCTCTACAGAAGGCGTTTCC
ACAATTTGAGATCGAAGTAGAGCAGGTCACACCGAATGACCATGCTAACGCGAGGGCGTTTT
CGCACCTTGCCAGTAAACTAATTGAAGGAGAGGTGGAAGTGGGCACAACCATCCTGGACATA
GGCAGCGCGCCGGCCAGGAGGATGATGTCCAAACATGCATACCACTGCGTCTGCCCACTGAG
GAGTGCAGAAGACCCAGAACGGTTAGCGGGATACGCGAAGAAGCTTATGAGTGCAGCCGGA
AATGTTACTGATCTCAACATCTCGGGAAAGATCACGGACTTGATGAACGTGATAGCTATACCC
GACCTTGAGACACCTACATTCTGCCTGCACACAGACCAGACCTGCCGCTATAATGCCGATGTG
GCCATCTATCAGGACGTCTACGCTGTCCACGCGCCCACATCGCTTTACCATCAAGCGTTAAAA
GGGGTCAGGGTCGTGTATTGGATCGGCTTCGATACCACGCCGTTTATGTTTGACGTAATGGCG
GGAGCGTACCCAAGCTATTCTACGAATTGGGCAGACGAACTGGTGCTACAGGCCAGAAACAT
CGGCCTGTGCAGCTCCAGATTGAGTGAGGGGAGATATCTGCAGAGGGCNATCCATTATGAGA
AAGAAAGCCCTTAGACCAAGCGACCGAGTTATGTTCTCGGTAGGTTCGACGTTGTATACGGA
GAGCCGCTCGCTACTGAAAAGCTGGCACCTGCCGTCGACTTTTCATCTAAAGGGTAAGGACTC

CTACACATGTAGATGTGACACCATAGTCAGTTGCGAGGGATACGTCGTAAAGAAGATCACGA
TGAGCCCAGGCCTATTCGGGAAGCCAGTACCGTACGCCGTCACCCATCATGCCGACGGCTTCC
TCGTGTGCAAGACCACGGACACGGTCAAAGGGGAGAGGGTTTNCTTCCCCGTATGTACGTAT
GTCCCAGCGACGATTTGCGACCAGATGACTGGGATCTTAGCCACGGATGTTACGCCTGATGAT
GCTAAGAAGTTACTCGCTGTGCCTAGAACCTGCGGAGCTCNTGCAGATATCGGGCGCACGCA
GAGAAATGTAAATACTATGAAAAATTACCTGCTACCTGTGGTCGCCCAGGCCTTCAGCAAGT
GGGCGAAAGAGTATAAAGCCGACTTGGAAGACGAGAAACCGCTGGGGGTCAGAGAACGCA
ACCTGACCATGTGTTGCTTGTGGGCCTTCCGAACTAGGAAGACCCACACCCTGTATAAGAAGC
CGGACACGCAGACTATAGTGAAAGTCCCTTCTGAATATACCTCCTTACTCATCCCGAGTCTAT
GGACGAGCGGGATCTCCATGACTCTGAGGAGACGGCTGAAAGTGCTGCTGACTGCCCGACTG
AACCAGAAATTGACGTGCCCTTTGGACGCGGTGACAGCAAAGATTGCAGAGGACGATGATCG
TGAGGCGCGTGAAGCTGAGTTAACCAGAGAGGCGCTACCTCCTTTGACTGCTACCGAGCCAA
CCGGCGACGATATACAGGTCGACATCGAGGAGCTTGACGCTAGGGCGGGTGCGGGAGTAGTG
AACACACCCCGCAACGCCGTCCGAGTCACCGCTCAGAGTGGTGACCTGTTGGTTGGCAGATA
TCTGGTGCTCAGCCCGCAGACTGTATTGCGCAGTGACAAACTACGCTTGATCCACGAACTGGC
AGAACAAGTGAAGATTATCACGCATTCGGGTAGGGCCGGTAGATATCCTGTTGAAGGCTATG
ACGGACGTGTGCTGGTACCTACTGGCAGCGCTCTGAGTTTCGCCGATTTCCAGGCGCTTAGTG
AGAGTGCCACAATGGTATACAACGAGCGAGAGTTCATGAACAGGAAACTCTACCACATTGCC
ACGCACGGCCCGGCCCTGAACACAGACGAGGAGAACTACGAGAAGGTGAGAGCGGAAAGA
AGCGACGCCGAGTACGTGTTCGACGTGGACGCACGGAAGTGCGTCAAACGCGAGCAGGCGT
CGGGAATAGTGTTAGTGGGAGACGCGATCAATCCACCTTACCATGAGTTCGCCTTTGAAGGCC
TAAAAACACGCCCTTCGGTGCCCTATAAAGTACCGACTATCGGTGTGTTTGGAGTTCCGGGAT
CAGGCAAATCGGCCATTATTAAGTCTGTGGTCACCAGACGTGACCTAGTCACTAGCGGCAAG
AAAGAGAACTGCAATGAGATCATGACCGACGTCAAGAAGCAGAGAGGATTGGATATCGTAG
CCAAGACGGTGGACTCTATCCTATTAAACGGTTGCAAACACAGCCCTGAGGTGTTGTTCGTAG
ACGAGGCGTTTGCCTGCCATGCAGGCACTCTGCTCGCGCTCATTGCGATCGTGAGACCCACCA
AGAAAGTTGTGTTATGCGGCGATCCGAAGCAATGCGGCTTCTTTAACATGATGCAATTAAAAG
TCAATTACAATCATGACATATGCACTGAAGTGCATCACAAAAGCATCTCTCGCCGATGCACA
ATACCGGTTACATCCATTGTCTCTACACTGCACTACGGAGGCAGGATGCGCACTACGAACCCG
CGCAACGACCCTATCGAAATTGACATCACCAGTAGCACCAAACCCAAGCCCGGAGTCTTGGT
TTTAACATGTTTACGTAACTGGGTTAAACAGCTGCAGTTGGACTATCGTGGACACGAGGTCAT
GACTGCGGCAGCGTCGCAAGGCTTGACCAGGAAAGGAGTATATGCAGTGAGGATGAAAGTC
AATGAGAACCCACTATATGCTCCAGCTTCCGAACACGTCAATGTCTTGTTGACACGCACGGAG
GGAAGGCTAGTGTGGAAAACGCTGGCCGGAGATCCGTGGATCAAGGTGCTAACTAACGTACC
CAAAGGAAACTTCACAGCCACCATTGACGAGTGGCAGGAGGAGCACGACAACATTATGAAC
GCCATAAGAGGGGAAGTGGCGCTGACCGACCCTTTCCAAAACAAAGCAAATGTTTGTTGGGC
AAAAGCTCTGGAACCAGTCCTTGCTACCGCTGGCATCCGACTCAGTGCAGCGGAGTGGAGCG
ACTTGATAGTCGCTTTCAAGGAAGATAAAGCATANTCGACCGAGGTCGCGCTAAATGAGATC
TGCACCAGAATGTACGGGTTTGACCTAGATAGCGGCTTGTTTTCGGCTCCGCTGGTATCCATGA
GATACGAGAACCATCACTGGGACAACTCACCGGGAGGTAAGATGTATGGATTCAACACACA
GGCGCGCCAGCAGGCCGGAGCTCTGCAGATATCCCTTCCTGCAAGGAAAATGGAGACTGAAC
CAGCAGATCCTTTTATCGGAGAGGAGGACGCAACCAGTGAGCTCATCCGCTAACATAGTTCC
CATTAACCGTAGGCTGCCGCACACTCTGGTGCTGGAACACGTCAAGCTACCCGGGGACCGCG
TAGAGCGGATCGTGATGCAAATTCGCGCGCATCACGTGCTGCTAGTGGGTGAGTATAACCTGC
ACCTGCCTTTACGGCGTGTTACCTGGATCGCCCCTCCCTATATACGGGGCGCAGACCGCATCT
ACGACCTGAGCTTAGGGTTGCCAACTGACCTGGGCAGGTATGATCTGGCGTTCATAAATGTAC

ATACAGAATTCCGCAACCACCACTACCAGCAGTGTGAGGACCATGCAATGAAGTTGCAGATG
CTAGGAGGTGACGCCCTACGCCACCTGAAACCGGGCGGATGCCTGCTGATGCGCGCCTATGG
GTACGCAGACAGGACCAGTGAGATGGTGGTGAATGCGCTAGCGCGTAAATTCGCATCAATAC
GCGTACTGCGACCAGTGTGTGTGGCCTCTAACACTGAAGTCTTACTGCTGCTGTCGGGCTTTGA
CAACGGGAAACGCCAACTGACAATGCACACATCCAACCTGAGACTCTCCAGCGTTTACTCGG
GCAACGCACTACACACGGCAGGGTGCGCACCATCATATAGAGTCGTAAGAGGCAATATCACC
GACTCCGACGCCGACGTGCTAGTAAATCAGTTGGGCGTGAACAACAGGGTCTGCGACGGAGT
CTGTAGGGCCATGGTCAAGAAGTGGCCTTCCTGCCTACCAACGACAACTGGAAAAGTAGGCG
ACGCCGTACTGACCACTGAGCCTCGAAAAATCGTGCACGCTTACTGTCCTAATTTTGGAACCA
GTAGAGAGGAGGTTGCCGATGCCGACCTAGCAGCTGTGTATAGAGCCGTGGCGTCCTTGGCT
GACGAGACAGTCCGCACAATGGCCATACCACTCCTGTCAACGGGGACGTTCGCTGGGGGAAA
GGACCGCGTGTTGCAGTCGTTGAACCACCTATTTACGGCCCTGGACACCACGGACGTCGATGT
AACGATATACTGCCGGGATAAGTCGTGGGAAAAGAAAATCCAAGAGGCCATTGATATGAGG
ACGGCAACCGAACTGCTAGATGACGACACAACGGTGATGAAAGAGCTAACCAGGGTGCATC
CTGATAGCTGCCTAGTGGGGCGCAGTGGATTCAGCACGGTGGACGGACGGCTGCATTCGTAC
CTTGAAGGAACTAGGTTCCACCAGACTGCTGTCGACGTGGCAGAAATAGCCACTCTGTGGCC
AAGGAGAGAGGAAGCGAACGAGCAGATAACACACTACGTCCTCGGCGAATCCATGGAGGCC
ATAAGAACCAAATGCCCGGTGGATGATACCGACTCGTCGGCACCACCATGCACCGTCCCGTG
CCTATGCCGCTACGCCATGACCCCCGAGCGCGTACACAGATTGCGCGCCGCGCAGGTGAAGC
AGTTCACAGTCTGCTCCTCGTTCCCGCTGCCAAAGTACAAGATACCAGGCGTGCAGAGAGTG
GCGTGTTCGGCTGTAATGTTGTTTAATCACGACGTCCCAGCGCTGGTAAGCCCTCGCAAGTAT
AGGGAACCGAGCATTAGCAGCGAGTCGTCATCCTCTGGACTGTCTGTGTTCGACCTGGACATA
GGCTCTGATTCAGAGTACGAACCAATGGAACCCGTGCAACCCGAACCGCTGATTGACTTGGC
AGTCGTAGAAGAGACAGTCCCCGTCAGACTGGAACGGGTGGCCCCTGTGGCTGCACCTCGCA
GAGCCCGCGCGACGCCCTTTACTTTGGAGCAGCGGGTTGTAGCACCGGTTCCTGCGCCGCGTA
CTATGCCAGTCAGACCCCCTCGCCGGAAGAAAGCGGCGACCAGAACACCTGAAAGGATTTCG
TTCGGCGATTTAGATGCCGAGTGCATGGCCATCATAAATGACGACCTGACTTTCGGGGACTTC
GGCGCGGGCGAGTTCGAACGATTAACGTCAGCATGACTAGACCGGGCGGGGGCCTACATATT
CTCATCGGATACAGGCCCAGGACACCTACAGCAAAGATCGGTCAGGCAAACCAGACTAGCG
GACTGCGTGGCGGAGGACGTACATGAAGAGAGAGTATTCGCCCCGAAGTGTGACAAGGATA
AGGAAAGGCTGCTGCTTTTACAAATGCAGATGGCACCTACAGAAGCCAACAAAAGCCGCTAC
CAATCCAGGAAAGTGGAGAACATGAAGGCAGAGGTTATCGACAGACTGTTGGGCGGAGCGA
AATTGTTCGTGACACCCACAACCGACTGCCGATACGTGACACACAAGCACCCAAAGCCGATG
TACTCGACTAGCGTAGCATCCTACCTGAGTTCGGCCAAGACTGCAGTGGCAGCATGCAATGA
ATTCTTAAGTAGGAACTACCCAACTGTGGCATCCTATCAGATCACGGATGAGTACGACGCCTA
CCTGGACATGGTGGACGGTTCCGAGAGCTGTCTAGACAGAGCAGCCTTTTGTCCGTCNAAATT
ACGCAGCTTTCCGAAGAAGCACAGCTACCATCGAGCCGAAATAAGAAGTGCCGTTCCGTCTC
CTTTCCAAAATACACTGCAGAACGTGCTGGCCGCTGCCACGAAACGCAACTGCAACGTGACG
CAGATGCGGGAGCTGCCGACCTTGGATTCCGCGGTGTTTAATGTCGAGTGCTTTAAGAGGTAC
GCATGCAACAACGACTACTGGGACGAATTTGCTCAAAAGCCCATCAGGTTGACGACGGAAAA
CATCACGTCCTACGTAACTAGACTGAAGGGCCCGAAGGCAGCCGCTCTATTTGCAAAAACCT
ACGACTTGAAGCCGTTGCAAGAGGTACCAATGGACCGCTTCGTCGTGGATATGAAGAGAGAC
GTGAAGGTGACACCCGGCACTAAGCATACAGAAGAACGGCCAAAGGTGCAAGTCATACAGG
CAGCCGAACCCCTAGCCACCGCCTATCTGTGCGGTATACATAGAGAACTGGTTAGAAGGTTA
AATGCAGTTCTGCTGCCGAACGTCCACACCCTGTTCGACATGTCGGCGGAAGACTTTGATGCT
ATAATATCCGAGCACTTCCGCCCTGGGGACGCTGTACTAGAAACAGATATCGCATCGTTCGAC

AAGAGCCAAGACGACTCGCTGGCGTACACGGGGCTGATGTTGCTAGAGGATCTCGGCGTCGA
CCAGCCTTTGCTCGAGTTGATCGAAGCGTCGTTCGGGGAAATAACAAGCACGCATTTACCAAC
GGGCACTAGGTTCAAGTTCGGTGCCATGATGAAATCTGGCATGTTCCTGACGCTCTTCGTGAA
TACGATGCTCAACATGACTATAGCTAGCAGAGTGTTAGAAGAACGGCTGACCAATTCCAAAT
GTGCCGCCTTTATCGGCGATGATAACATTGTGCATGGAGTGAAATCTGATAAACTGCTTGCTG
AGAGATGCGCCGCATGGATGAACATGGAAGTGAAGATCATCGATGCAGTCATGTGTGAGCGC
CCCCTACTTCTGCGGAGGGTTTATCGTGTTTGACCAAGTTACAGGTACCTGTTGCAGAGTGGCG
GACCCGCTGAAGAGACTCTTTAAGCTCGGAAAACCGCTGCCTGCTGAAGACAAACAGGACGA
GGACCGTAGAAGGGCGTTGGCCGACGAGGCACTACGGTGGAACCGCGTAGGTATCCAAGCA
GACTTGGAGGCCGCAATGAGCAGCCGTTACGAGGTCGAGGGGATCCGAAACGTCATCACGGC
GTTAACCACGCTGTCGCGGAATTATCACAATTTCCGCCATCTAAGAGGACCCGTTATTGACCT
CTACGGCGGTCCTAAATAGTTGCGTGAATACATATTCTAATAACAGATATTATTGACGCAGCA
CCATGAATTACATACCTACGCAGACGTTCTACGGCCGCCGATGGCGTCCTCGCCCGGCGGCCC
GCCCCTGGGTGGCTCCACCACCCGTATACTATCCGCCGCCACCACCCGTGCCTGTCGACCCGC
AAGCGCAGCAAATGCAACAACTTATTGCTGCGGTCAATACGCTGGCTATAAGGCAGAATGGC
ACCCGAACACCTGGACAACAACGAAGGAAACGTCAACCAAACAAACCAAAGAGGAAACAG
ACACCCCCAAAGAAACAGAACCCGGCGAAAACAAAGAACAAGCAGAAACCGCAACCACCC
AAGCCTAAGAAACGGAAACCCGGCAAGAGAGAAAGGAAATGCATGAAGATAGAGAATGAT
TGCATATTCGAGGTCAAGCTCGAAGGCAAGGTCACTGGGTACGCCTGCCTGGTAGGAGATAA
AGTGATGAAACCAGCACACGTGAAAGGAGTCATAGATAACCCTGACCTTGCCAAGCTTGCTT
TTAAGAAATCGAGCAAGTATGACCTTGAGTGTGCGCAAATTCCGGTCCACATGAAGTCAGAT
GCCTCGCAGTTCACCCACGAGAAACCAGAAGGTCACTACAACTGGCACCATGGTGCAGTACA
ATACCTGAACGGAAGATTCACCATCCCGACAGGCGCTGGGAAGCCAGGGGACAGCGGTAGG
CCTATCTTTGACAACAAGGGTCGCGTAGTGGCCATTGTGCTGGGGGGAGCCAACGAGGGAGC
GAGGACGGCTCTATCGGTTGTCACCTGGAACAAAGACATAGTTACGCGCATCACCCCAGAAG
GAACTGAGGAGTGGACTGCCCTGGTGACAACTGCTTGCATCTTGAGCAATCTGACTTTCGACT
GCAGCCTGCCACCATGTGCGCCTTGCTGCTATGAAAAAGACGCAGAGGGCACCCTGAGGATG
CTAGAGGACAACGTCGATAACCCCGGATACTACGATCTCCTGGCTGCATCAACGCATTGTGAC
GCCCCGCAGCGGCGTCGCCGCAGGGGGCTAACTGAGGACTACGAGGCTTACAAACTCACTAA
GCCGTACATAGCCTATTGCTCTGACTGCGGGAATGGACAGTTTTGCTACAGCCCGATAGCTAT
CGAGAGAGTCAGGGCCGAGGCATCGGACGGAATGCTCAAGATACAGATCTCTGCGCAAATA
GGCCTGCAGGTGGACGGAACTCATTCGTGGACGAAAATCAGATACATGAAAGGGCACGACG
TGGAGGACACGGACAGGAACTCGCTGGAGGTGTTCACCACCGGAGAGTGTACGGTCCATGGC
ACCATGGGGCACTTCATCGTAGCTACATGCCCCGAAGGTGACTCCTTGACAGTGGCGTTCGTT
GATAAACATAAGGTCAGGCACGCTTGCAGGATAGCATATAAGCATCGTGTCCCCGTACTGGG
CAGAGAGCACTTTACGGTACGGCCACATCATGGAGTAGAATTGCCATGCACCACGTACGCCA
TGAGAACATCAGTCACTACCGAAGAAATAGAAATGCACGTGGCGCATGACGTGCCCGACAA
CACCTTTTATCCAAGACCGGAAACAAAGTGAAGATAACGCCAAAAGGAAAGTCTATTCGCTA
CAACTGCACGTGTGGGTCTAAGGAGAGCGGTGTCACAAAGCAAGACAAAGAATTTGACAACT
GCGAAGTTTCGCAGTGCCACACCATGGTGACCGCCCACGATAAGTGGCAGTTTAACTCTCCTT
ATGTCCCTAGGGCAGGCTCAGGCAAGAAAGGAAAGATCCACGTACCCTTCCCACTGAGCAAC
TCTACGTGCAGAGTTCCGTTGGCGCCTTTACCGAACACCATCCCGGCAAAGAATGGAATCACA
TTGCAGTTGCATCCGGTCGCCCCGACGCTACTTACCTACCGCACCCTCGGAGAGAAACCAGA
ACACCACACAGAGTGGATATCAGAAAGTTGCGAACGTACACTCCCCGTACCTGAGGAGGGGT
TGGAGTACACATGGGGCAATCATGCCCCCGTGAGACTATGGGCACAACTGACGACTAAGGGT
TCAGCCCATGGGATGCCGCACGAAATCTTCTCATATTACTACGGATTGTACCCTGCCGCGACG

GTTGCAGTGTGCGTGGGGCTAGCGTGTGTGATCTTGCTGGCCCTGTCCGCGTCCTGCTGCCTGT
GCGTGTCAGCGAGAAATAAGTGCTTGACCCCGTACGCGTTGACGCCAGGGGCCGTGGTGCCG
TGCACTTTGAGCTTATTGTGCTGCGCCCCCAGAGCCAAGGCCGCAACGTTTGCGGAGACAGCG
GCATACCTATGGGACGAGAACCAGACGGTGTTCTGGATGCAATTCGCAATCCCCGTAGCATG
CTTTATGATAGTGACATATTGCCTGCGCCACTTGATGCTGTGCTGTAGGACCGCTTCTTTTTTAG
TGGCAGTAAGCCTGGGAATGGGGGCGATATCTGCAGAGCNTCTGAGCATAGTGTAANNCTAG
GCACAGCGAGTCTTGGTTTTAACCTTNCAGANCAATACGTAACTGAACGAATCGTTCAGTTAC
GTATTGCTAAGGTTAAAAACCAANNNGACTCGCTGACCCTAGTTCAGCACCACGGAGAGAGC
NCTGCANGATATCGAGACCTGAAAATGATCCTTGGTCCCATATCCACCGCGTGGAGCCCCTTC
GACCCAAAGATCGTCATCTACAAGGACGAAGTCTACAATCAGGATTATCCACCGTACGGATC
CGGGCAACCGGGTAGATTTGGGGACTTACAGAGCAGGACCACCGAGAGTAACGATGTGTAC
GCCAATACTGCACTGAAGCTGGCTCGTCCATCTGCCGGCACGGTGCATGTTCCATATACCCAG
ACGCCGTCCGGGTTTAAGTATTGGCTAAAAGAAAAAGGGGACGCATTGAACCATAAGGCTCC
TTTCGGCTGCATCATCAAGACGAACCCCGTAAGGGCAGAAAATTGTGCAGTCGGAAACATAC
CAGTGTCTCTAGACATTCCCGACGCGGCTTTCACACGCATAGTCGACGCACCATCGCTAACCG
GCCTGAAGTGCGAGGTGGCGACTTGCACGCACTCATCGGACTTTGGAGGCACTTTGGTGGTGG
AGTACAAGACCGACAAAGTGGGGACGTGTGCCGTCCACTCAGAATNCAACACGGCCGTTATG
CAGGAGACGAGTCTGTCCGTGACGATGGACGGCCGAGGCACGCTGCATTTCTCCACCGCCTC
AGCCTCACCGTCCTTCGTACTGAAAGTGTGCAGTAGTAAAACCACTTGCACAGCAAAGTGCGT
GCCGCCGAAGGATCACGTCGTCCCTTTTCCTGCCAACCACAACAATGTTGTGTTNCCGGACAT
TTGCAGTACTAGGCACAGCGAGTCTTGGTTTTAACTCCACCCATACGTAACTGAACGGAGCTA
CTGTGGTGATTGCTGTGGGATCACCATATTCTTAATAGTTACTTGCATGGCTTTTAGTAGCCAC
TAGA

>Wesselsbron virus (animal origin)

AGTATATTCTGCGTGCTAGTCGTTCGACGTTAGTCCGTGGAGTGAGCTTCTATTAGAGTCGTTA
ACACGTTTGAATAATTTCTACTGAAAGGAGTAGAAGAAAGGAGATTCATTCCCAATGGCAAC
AAAGGGGATGAATAAGTCTCGGGCTCGATCCCGAGGCGTCAATATGGTAGCGGCTAGAGTGA
AGAACCTAGCCGTTAAAGTAAAAAACAAAACAAAACAAAGTGCAAGAGGTTTGCGAGGGTT
CCTCTTGTTTCTAGTGGCCCAAATTTTCTGGGCTAGAAAACTAACCCCGCAGGTTAAAAGATT
GTGGAGAATGGTGGACAAAGTGCAAGGTCTCCGGATCCTGAAGAACATTAGGAACATCGTTA
CCAATCTGATGAAGGGACTGGCTGGTCGCAAGAAAAAGCGAAGCTTGACAGTACCCTTGGTC
CTTTTGCTGATTCCACTGATTGCTTACTCAGCTACTGTCACCCGTCAACGCGGATTGGGGCTTTT
GCTCAACGTCACTTTTGCTGACGTTGGGAAGACCTATGAGGTTGAGGGGGGCAATTGCTCTGT
CAACACTTTGGATGCGGGCAAATGGTGTGATGATTATGTGGAGTATGAGTGCGTCACTCTCTC
TGAGGGTGAATAACCTGACGTAACTGGAATGTTGGTGCTACGGAGTGGACAATGTCCGTGTC
ACCTATGGAAGATGCAAGAGTGGTGGTTCACGAAGATCGAGGCGCTCTGCCGTCATAACTCC
ACATGTAGACAAAGGACTTACTACTAGACAGGAAAAGTGGCTGCCCACAAAGATTGGAGAA
CAGCAACTGCAGAAAGTGGAAAAATGGATTATGAGGAATCCTCTCTATGCACTTGGGGCGGT
GGCTTTAGCATACTTTGTTGGCACATCAAATGTGCAGAGGGTAGTTATTGCCATCTTGCTTCTT
GGCATTGGACCAGCTTATTCAACACACTGCTTAGGAATTCCAAAAAGGGACTTCATAAGGGG
ACTTGATGGAAACACCTGGGTGTCAGTGGTTTTAGAACAGGGCAGCTGCGTGACTTTGATAGC
CGACAACAAGCCATCAGTAGATATTTGGCTGAGTTCCATAGTGGTTGATACGCCAACTCTTGT
GAGGAAAGTTTGCTATGCGAGCTCTGTGACTGGATCGAAAGCAACTGGAGCCTGTCCAACTA
TGGGAGACGCCCACATGTCTGAAGAAGGAAATGAAGAATGGGAATGCAAAAGATCATACTC

GGACCGAGGATGGGGTAACGGTTGTGGGCTTTTTGGTAAAGGAAGCATTGTGGCATGCGTAA
CTAATTTTCATGCACACATGAAATGGAGGTTTATCAGATAGATGCCACTAAGATTGAATATAC
CATCAGTGCCCAAGTTCACTCGGGAGCCAAGAAAGATGATTGGGTAAATCACACAAAATTGG
TGACTTTTGTTCCGACCACAGGAACATCAACTGTTGCTTTTACAGGCTATGGAAACTTTGGCTT
AGAGTGTCACGTTCAGATGATGGTGGACCTCAGCAATTCCTATCTGGTGAAAGTAGGAACTGA
TGCCTGGCTGGTCAACAAACAATGGGTTCACGATATTACTCTCCCATGGCAGAGTGGAACAA
GACGACATCTGCAGAGCTCCGGCAGGTCATGGTTGACTTGGAATGAACCTTACTGATATCTTA
GCAGAGTCACTGATGAACGCGCTAATGTCACTAGCAATACGGAACCGAACGAACTTTCTAAC
CTTAGCAATACGTAACTGAACGAGCTAAGGTTAAAAGGATTCATTCCCAATGGTAACACCAG
CACCTGCCGGAGCTCTGCAGATATCATGGCGGGAACAGAAAACCGTGGAAGGTCATCACAAC
CAACTTATTGCTGTCAAACAATGATGAAGTGTTGCGGAGATCATTCACCATTTGAGAGAGTTA
CATCATCGTTGGTAATGGAGATGATAAACTTTACATACCACTGGCAAAGATCAGGAAGCACC
ATCGGGAATCTGTTCAGAGACAATGAAAGGAGCCCAACGCATGATTATCACAGGTGAGCACT
CGTGGGACTTTGGCTCAACTGGAGGATTTTCTATCCATTGCAAAAGCTGTGCACACAGTCTTTG
GTGCTGCCTTCCATGCAGTTTTTGGTGGTCTCAGTTGGATAACCAAGATATTGATTGGAGGGTT
GCTCATCTGGCTGGGTCTCAACTCGGAGAAGTTCAATGTCAATGGGCTTCATATGCATTGGAG
CCTTGCTGCTTGTGCTGGCCACAGGAGTTGAGCAGAAGTGGGTTGCTCCTCGAGTTGGAAGCA
CGAAATGAAATGTGGGGATGGTGTTTTTGTCTTTAAGGATTCTGATGATTGGTTTTCCAAATAC
TCAGTGCTACATTCCAGAAGATCCAAAACAATGGCCACCTTGATTCATCAAGCCCACCCAGG
ATGGACTATATGTGGTTTGAGTTCTGTCAGCGACCTGGAACATCGAATGTGGTATTCTAGAGT
CGACGAGATAAACGCCATCTGATGAAAATGAAGTGGATCTCACAGTGGTTGTCCAAGAATCA
GACGTACTAAGTGAGGATCGCATGCTTCCTCGGCCAAAAAGTGAATTGAAGTATGGATGGAA
GACTTGGGGAAAAATATCATCTTTAATCCATCTAGAAAAAATGGTACATTCATCATTGATGGA
AAAGGAAGCAGAATACCCTCTTCAACAAGAGAGTGTGGAACTCAATACGGGTTGAGGAGTTT
GTTACAGGAGTTTACCAGACAAGAGTCTTCATGAGACCGGAATTTGATTACACCAAACTGTGT
GACACGGGAACTTTGGAGCTGCTGTTAAAGGTAGCGTTTCAGCTCATGGAGACCCCATGTTCT
GATGGAATCAGAAGAGATCAATGGCACATGGATGATCACGACCTTGGAAGTTATTTCAAATT
ACCGTGAGTGGAATGGCCTAGTCTATTACCACACTGGATGGAGCAAAAGTGGTGGAACTAGA
CATGTTCAGTTACGTATTGCCCAGGAGCATTGCTAGGATCCATTCACAGTAANCACCAGCACC
TGGCTCAGGATATCAAAGGTGCAAACTAGTNACCTATGGCACAATGTCCCACTTGAAATAAA
ANNAGAAGAGTGCCCAGGCACCACTGTTGTGGTAGATGAGAAGTGCGATGATAGAGCAAAG
TCAGTAAGATCAACAACAGATAGTGGAAAGATCATACCTGAATGGTGCTGCCGCAGTTGCAC
CATGCCTCCAGTGAGTTTCTNGGGGCCTGACGGCTGCTGGTATTCCATGGAAGTTAGGCCAAA
ACACACCAATGAAGCACATCTGGTGAAATCCTGGGTTGTTGCTTCCAAAGGAGATGTTGACCC
TTTTCTCTGGGGCTTCTCATGTTGTTTCTCTGCAGCGACATGTTCCTGATGAAGAGGTTCTCCAT
GAGACGTATTGCTAAGTTGAAAGTCTNGTCATGCTGGGAGCGATCACATTCCGGAGCTCTGAG
ATATCTGGATCTCCTAAGGTATGCCATAACAGTTGGAATGTACATGGCTGAGATAAACAGTGG
AGGAGATGTCACACATCTGGCTCTGCTAGCGGTTTTTCAGGTGCGGGCTGGCTTTGTTAGCATG
CTGGCCCTAAAACGACTTTGGAGCCCTAGAGAGGGNNTTGTGGCAACATGTGGAATTGTCAT
GGTCCAGCTTGCATTAGGAGACATATTGTCAACTGACATCATGGAATGGCTGAATGCGGCCG
GTATGGCAGTGCTCATCATCAAATCAATTGTTGAACCTAAACGGTGTAATGCTGTGTTGCCTTT
GCTTTGCTTGCTGACCCCACTGACTGTGGCCGAAATACAAAGGGCAGTGATGTTTGTCTGTTCC
ATTGTGATTTTTGTCACAGTTTGGCAGACTGACAGCGTCTCAACCAGNAAAACCATNCCCTTG
GTTGCTCTGACTGTTTGCTCTTTCTTCAAATGGACTAGCCCCTTTTTAGGCATAGTTTGCTATCTT
GCATTCACACGCCTTCCCCAGCGATCTTGGCCTCTTGGAGAGACCATGGCTGCTGTTGGGCTG
GTTGGAGTGCTTGCTGGCATGGGACTGAAGGACATGAACGGAATGCTTGGACCAGTGGCCGT

TGGTGGAGTGTTACTAATTGTAATGAGTCTTTCAGGAAAGGTTGATGGGCTGGTAATCAAGAA
AGTGGCTGACGTGACCTGGGATGAGGATGCTGAGATTAGTGGAGCATCACACCGTTATGATG
TGGAACAAACTGACACAGGTGAATTCAAATGTTACCGTGAAATGAAGAACNNGCTGATCAGT
CCTGCAGTAGCTCAGGTGGGAATGAATCCTTTTGGCATTACCCTGGGAATGAATCCTTAGCAA
TGTAAACACTGAAGCGAAGTACACAGTTACGTATTGCTGGGTTAAAGTTCATTCCCACGGTAA
CACAGCACCTGCCGGAGCTCTGCGGANNNTATCGGGCCCGGCCAGTATTGCAGCAAGAGGTT
GGAGCAACCCACAGATCCAGGGCTCGGNNGTGTGCCACCATATTCATGTCAGCTACCCCTCC
TGGGACAAGCAATGAATTTCCAGAGTCAAATAGCATGATTGAAGACGTCAAGAAAAATGTTC
CCTCAGAACCATGGACAAAAGGGCATGAATGGATTTTAGAGGACAGACGCCCTACTGCTTGG
TTTCTCCCCTCCATCAGGATAGCCAATTCTATCGCAAACTGCCTCCGTAAGGCTGGACAGAAC
AGTGGTGGTGCTGAATAGAAAGACCTTTGAAAAAGAGTATCCCACAATAAAGTCAAAAANG
CCTGACTTCATACTGGCCACTGATATAGCTGAAATGGGAGCCAACTTGAAAGTGGAGCGAGT
CATCGACTGCCGCACAGCTTACAAGCCAATTTTGGTTGATGATGCCACGAAAGTCATGGTCAA
AGGGCCCCTGCGCATATCAGCCTCATCAGCTGCACAGCGGCGTGGAAGGATTGGGAGAGATC
CCAACAGAGACACTGACACCTACATTTATGGAGACTCAACAACTGAGGACAATGGGCACTAT
GTCTGTTGGACAGAAGGTTCAATGCTCTTGGACAATATGGAAATCAGGAACGGCATGATCGC
CCCGTTATACGGAGTGGAAGGCACCAAAACAACAACAAGTCCGGGAGAAACACGACTAAGA
GAAGACCAACGGAAAGTCGTTCCGTATTGCTGGTCAAGTTACTTGACATGCCGGTTAGGTTAA
TTCATGGTACAGCACCGCGGAGCTCTGCAGATATCAAGGTGCAGGACAGAAGCTGGTGTTTTG
ATGGTGAGGATGACAACACCTTACTTAATGACAATGGAGAGCCAATTTTAGCCAGAAGCCCA
GGAGGAGCCAAAAAACCGCTGAAGCCCCGCTGGGTCGACACCAGGGTATGCAGTGACAATG
CATCATTAATTGATTTTATCAAATTTGCTGAAGGCCGACGTTCTGCTAGCGGGATTTTGTTAGG
CTTGCAAGGATTTCCTGAATTTCTGTCTGGGAAAATGCGGGAAGCGATCGACACAGTAACAGT
CCTATACACTAGTGACACTGGAAGTAGAGCCTACAAACACGCACTAGCAATGATGCCCGAAG
CTACAACAATTTTCCTACTGGTGATGCTGGCTATCATTTGTACATCTGGGGTCATCATGTTTTTT
TTGGCTCCAAAAGGACTAAGCAGGATGTCAATGGCAATGATGACAATGCTGGTGTCTGCCTAT
CTTATGTCATTGGGAGGAATGAACCCTGTCCAGATTTCTTGTGTCATGTTGGTATTTTTCATTTT
CATGGTGGTGCTAATTCCAGAACCGGGCACACAGAGATCTACCTATGACAATCAAATCATCT
ACTTGTTGGTAGGGGTGCTGAGCTTAATTCTCTTAGTGGCCGCAAATGAAATGGAACTGCTGG
AAAAAACAAAAAGGGATATTTTTGGAGCTGTGGTGGTCGAGGAGGCTAAAAGATGGACATTT
CCTGAGTTCGACCTACGCCCAGGAGCAGCCTGGACTGTCTACGTCGGGCTTGTTACTCCTGGT
AACCCCATGCTCCACCATTGGATAAAAATTGATTACGGGAACATTTCTCTGTCTGGCATAACA
CAAAATGCACAGGTGCTGGGACTCATGGATAGAGGCATACCCTTCATCAAAATGAACATGTC
AGTGGTCATTCTCCTCCTGAGTGCATGGAATGGAATAACTCTGCTACCACTTTTCGCAGGTATG
GGAGCAGCTGCACTCCATTGGGGTTTCATCCTACCGGGACTCCGAGCACAGGCCGCTAAAGC
TGCACAAAAAAGAGTTTACCATGGGGTGGCAAAGAATCCAGTGGTTGATGGGAATCCAACAG
TGGATATTGATGATGCTCCTGGCATGCCGGCCATGTATGAAAAGAAATTGGCCTTGGTGATTT
TGCTGGCGCTTTCAATCTTGAACTTGGTTTTGACCAGAACCCCCTTTGCAACGGCTGAAATGGT
TGTGCTAGGCTCAGCGGCGGTAGGACCACTCATTGAGGGCGACACCAATGCTTATTGGAATG
GACCTATTGCTGTAGCGTTTTCAGGATTGATGAGAGGAAACTACTATGCAACCATTGGACTTG
CCTACAATGGATGGTTGGCCAAACAGACAAGGAGAGGCAAAGCAGCTGGAGTGACCCTGGG
GGAAGTTTGGAAGAGGCAGCTGAACATGTTAGGGAAACAAGAATCTGAGAGGTACAAGGTG
CCAGACATTACTGAAGTGGACCGAACCGCAGCTATGCGTTACCTGAAAGAAGGACGAACAG
ATGTGGGAATCAGCGTCTCTCGAGGAGCAGCCAAAATCAGATGGCTTCACGAGCGCGGCTAC
CTCAGAATCACAGGACGTGTCCTGGACCTTGGATGCGGACGCGGTGGATGGTCATATTACGCT
GCCGCGCAGAAGGAAGTCATGAGCGTCAAAGGTTACACACTAGGAATTGAGGGTCATGAGA

AACCAATCCACATGCAAACATTGGGATGGAACATTGTCAAGTTCAAAGATAAGTCAAATGTG
TTCACCATGCCAACTGAACCAAGTGACACCTTACTCTGTGACATAGGTGAGTCTTCATCAAAT
CCCTTGGTTGAGAGGGACAGGACCATGAAAGTGTTGGAAAATTTTGAACGATGGAAACACGT
GAACACAGAAAATTTTTGTGTCAAAGTGTTGGCTCCCTATCATCCAGATGTGATTGAGAAGCT
GGAAAGACTGCAATTGAGGTTTGGTGGTGGAATTGTGAGGGTTCCATTTTCCAGAAATTCGAC
TCATGAAATGTACTACATATCAGGAGCCCGGAATAACATCACTCACATGGTCAACACAACAT
CAAGGAGTTTGCTAAGAAGAATGACTCGGCCTAGTGGGAAAGCCATCATAGAAGGGGACGT
GTTTCTTCCCACTGGAACGCGAAGCGTTGCCAGTGAAACAGGCACCATAGACCATGAGGCGC
TCAAGCTCAGAGTTAACCAGATTAAGGCAGAGTACTCAAGGACTTGGATACATGATTCAAAC
CTGCCATATCGAAAATGGCATTATCTAGGAATGAATCCTGTACCTATAGCAAAGCAACCGGC
AGTTCATCTTCAATGATCAATGGCATTGTCAAAGAAATGCTGTCGATGCCATAGGACAAATTC
AAATCTCGTGACTCTTCATGGCAAGTGACTCGACACAACCCCGTTTGGGCAGCAANGGGTGTT
TAAAGAGTAAAGTGGATTCACAGAAAGCCCCACCTACCCGACCTCTGCAGAGAACACGTGCG
ATCATGCGTGTTGTCAATGCTTGGTTGTTCCAACACCTCGCTCGTAAGAAGAAGCCCCGCATTT
GCACGCGTGAAGAGTTTGTGGCCAAAGTTCGTAGCCATGCCGCCCTTGGAGCGTATCTCGAAG
AGCAGGACAAATGGAAGAGTGCAAGTGAGGCTGTCCAGGATCCACAGTTCTGGAAACTGGTT
GATGATGAGAGGAAGCTGCATCTGCAAGGCCAATGCCGGACATGTGTGTACAACATGATGGG
GAAACGTGAAAAGAAACCCTCTGAATTTGGCAAAGCAAAGGGAAGCAGGGCGATCTGGTAC
ATGTGGCTAGGAGCTAGATTCCTTGAGTTTGAAGCACTGGGATTCTTAAATGAAGACCACTGG
GTGTCCGGAGAGAACTCAGGGGAGGGGTGGAAGGAACAGGCTTACAGTACCTTGGCTACATA
CTGAAGGAACTGGGTGGCAAAACAGGAGGAAACATGTATGCAGATGACACAGCCGGATGGG
ACACAAGGATCACGGAAGAAGACCTGGAGGATGAGCAAGAGATTCTAAAATACATGGATGA
AAAACACAAAAAACTGGCTTGGGCTGTGACGGAGTTGGCATACAAAAACAAGGTAGTCAAA
GTGATGCGTCCTGGCCCTGGAGGGTTGACCTTCATGGACATTATTTCAAGAAGGGATCAAAGA
GGGTCCGGGCAAGTGGTCACTTATGCCCTGAACACCGTGACTAATCTTAAGGTTCAACTCATA
CGCATGGCGGAGGCAGAGCATGTCATTACAAATTTTGATGTTGACACAGTTAGCCAGAAGAC
CCTGCAGGACTTGAGATGCTGGCTTGACAGATTTNGTNCAGATCGCTTGTCCAGGATGGCAGT
CAGTGGTGATGACTGTGTAGTGAAGCCCATTGATGACCAATTCGCCGATGCACTGACCCACTT
GAATTCCATGTCAAAGATTAGGAAAGATATAGATGACTGGAAACCATCCCAAGGCTGGGCCT
CGTGGGAGGATGTCCCATTCTGTTCCCACCACTTCCATGAACTGATCCTAAAGGATGGACGGT
CGATAATAGCACCATGCCGTGATCAGGATGAGCTCATAGGGAGGGCCCGTGTGTCCCCTGGA
AATGGATGGATGATCAGAGAAACAGCCTGCCTTAGCAAGGCATATGCACAAATGTGGCTGTT
GATGTATTTCCACCGCAGAGACCTTAGAGTTATGGCCAACGCAATCAACTCGACCGTTCCTGT
GGATTGGGTTCCAACTGGAAGGACAACATGGTCTATTCATGGAAAGGGAGAATGGATGACCA
CTGAAGACATGCTGCAGGTGTGGAACAGAGTTTGGATAGAAGACAATCCACATCAGACTGAC
AAAACACCAATCACTGAGTGGCGAGACATTCCTTATCTGCCAAAGTCAATTGACAAGACATG
CAACTCACTCGTTGGAACCACACAACGAGCTTCCTGGGCTAGAGATATCAAGCACACAGTCC
ATAGAATCCGTGGACTTGTTGGAAATGAAAAATATACAGACTATCTAGCCACTATGGACAGA
TTCAGGGAACTCGATGAGAGTGGCCCTGGAGAAGTCTTGTGGTAAATTCAACAAAATTTAAG
AAACCGGGATACAAACCACGGATCCAGAACCGGACTGGGTCACCTATTGAATAAAACCGGG
ATATAAACCACGGAGAGGACCGGACCTCTCACTCTGTAAAACCGGGATATAAACGAGCAAG
CAGGACCGGACCTGCTTTTAGAGTCAGCCCATCATTTGATGCCATGGCTAAGCTGTGAGGCCA
TGCTGGCTGGGACAGCCGCGACCACCCGCGCAAAGGGGTTCCTGGTAGGCTCAAACCAGGGT
AAAAAGTATTGGGAGCCTCCACCCACCGTTACCGCGCACGGTGGGAAAGATGGGGTCTAGAG
GTTAGAGGAGACCCNTNNCCTCCCGAGCACACATAGCGGACCATATTGACGCCAGGGAAAG

ACCGGAGACACTCCTTGATTCTGACCTTTCTAGCAATACGTAAAGAACAGACGAAACTGAATGCAGAGCTCCGGCAGGTGCTGGTGTT

Segment	Accession No.
L	NC_005301.3
M	NC_005300.2
S	NC_005302.1

Suppl. Table S3 Reference sequences used for the genome assembly (de-novo and map-to-reference)

3.1 CCHFV

3.2 RVFV

Segment	Accession No.
L	NC_014397.1
M	NC_014396.1
S	NC_014395.1

3.3 DUGV

Segment	Accession No.
L	NC_004159.1
M	NC_004158.1
S	NC_004157.1

3.4 NSDV

Segment	Accession No.
L	NC_034387.1
M	NC_034391.1
S	NC_034386.1

3.5 MIDV

NC_024887.1

3.6 WSLV

NC_012735.1
